# Simultaneous quantification of linezolid and its metabolites (PNU-142300 and PNU-142586) in oral fluid and capillary blood by UPLC-MS/MS: method validation and clinical application using non-invasive sampling techniques

**DOI:** 10.1186/s12967-026-08465-8

**Published:** 2026-06-17

**Authors:** Patricia González-Berdullas, Francisco Cajade-Pascual, Gonzalo Hermelo-Vidal, Iván Fernández-Castro, Sonia Molinos-Castro, Enrique Bandín-Vilar, Rosa María Roca-Sánchez, Nerea Vázquez-Agra, José Ramón Caeiro, Irene Zarra-Ferro, Elena Lendoiro, Anxo Fernández-Ferreiro, Cristina Mondelo-García

**Affiliations:** 1 FarmaCHUSLab Group, Health Research Institute of Santiago de Compostela (IDIS), Santiago de Compostela, 15706 Spain; 2 Pharmacy Department, University Clinical Hospital of Santiago de Compostela (SERGAS), Santiago de Compostela, 15706 Spain; 3 Internal Medicine Department, University Hospital of Santiago de Compostela, A Coruña, Spain; 4 Traumatology and Orthopaedics Service, University Hospital of Santiago de Compostela, Santiago de Compostela, 15706 Spain; 5 Toxicology Service. Institute of Forensic Sciences, University of Santiago de Compostela, San Francisco, s/n, Santiago de Compostela, 15782 Spain

**Keywords:** Linezolid, PNU-142300, PNU-142586, UPLC-MS/MS, Oral fluid, Capillary blood, Therapeutic drug monitoring, Non-invasive sampling techniques

## Abstract

**Background:**

Therapeutic drug monitoring (TDM) can improve the safety and efficacy of the antibiotic linezolid (LZD), particularly in patients at risk of toxicity or with high pharmacokinetic variability. Although oral fluid (OF) and capillary blood collected through microsampling have emerged as less invasive alternatives to venous blood, the applicability of these matrices for quantifying LZD and its major metabolites—PNU-142300 and PNU-142586—has not been established. This study aimed to develop and validate a UPLC-MS/MS method for the simultaneous quantification of LZD, PNU-142300, and PNU-142586 in OF and capillary blood collected with volumetric absorptive microsampling (VAMS).

**Methods:**

Method validation followed ICH M10 guidelines and included assessment of linearity, accuracy, precision, selectivity, matrix effects, hematocrit influence, dilution integrity, and short- and long-term stability. The method was further evaluated using paired clinical samples from 35 patients receiving LZD.

**Results:**

The assay showed linearity from 0.1 to 5 µg/mL for all analytes in plasma, OF, and VAMS, with accuracy and precision within ICH M10 acceptance criteria, demonstrating analytical feasibility across all matrices. In the clinical phase, LZD showed strong correlations with plasma in both VAMS and OF, supporting their feasibility for linezolid monitoring. However, metabolites showed strong but systematically biased correlations in VAMS, indicating that correction factors will be required before routine clinical use. In OF, metabolites were rarely detectable, precluding their monitoring in this matrix.

**Conclusions:**

Overall, these findings demonstrate for the first time the feasibility of using VAMS and OF as non-invasive matrices for LZD TDM. Regarding feasibility, LZD concentrations in both matrices correlated strongly with venous plasma and the method met all ICH M10 validation criteria. Regarding current limitations, direct clinical substitution is not yet possible for metabolite monitoring: systematic underestimation in VAMS requires prospective validation of correction factors, and the near-absence of metabolites in OF limits this matrix to linezolid monitoring only. Larger studies are needed to validate correction strategies and establish matrix-specific therapeutic targets prior to clinical implementation.

**Supplementary Information:**

The online version contains supplementary material available at https://doi.org/10.1186/s12967-026-08465-8.

## Background

Therapeutic Drug Monitoring (TDM) involves measuring drug concentrations in patient samples to guide dose adjustments. It is especially useful for drugs with narrow therapeutic windows or high pharmacokinetic variability, such as certain antibiotics. Linezolid (LZD) is a clinically relevant example where TDM can improve both safety and efficacy [1]. In clinical practice, trough concentrations of LZD in plasma or serum are commonly used as a surrogate for drug exposure, and are generally adequate for dose optimization [1].

LZD was developed by Pfizer and approved in 2000 as the first member of a new class of antibiotics: the oxazolidinones [2]. Structurally, oxazolidinones are five-membered cyclic compounds containing a carbonyl, an alkoxy, and an amino group in various positions.

Clinically, LZD acts as a bacteriostatic agent and is active against Gram-positive bacteria resistant to other treatments, including methicillin-resistant *Staphylococcus aureus* (MRSA) and vancomycin-resistant *Enterococcus* (VRE) [2]. It is also listed by the World Health Organization (WHO) as both an essential medicine and a last-resort treatment for multidrug-resistant tuberculosis (MDR-TB) [3]. Its mechanism of action involves binding to the peptidyl transferase center (PTC) of the bacterial ribosome, with its interaction at the A-site influenced by specific amino acid sequences, which ultimately leads to the selective inhibition of bacterial protein synthesis [4].

Notably, LZD has nearly 100% oral bioavailability and sufficient water solubility for intravenous use, allowing flexible switching between both administration routes [2]. It is available as an intravenous infusion solution and as oral tablets or suspension. It penetrates and accumulates well in many tissues, including bone, cerebrospinal fluid, and lungs, making it useful for treating infections at different sites caused by susceptible microorganisms [1, 5]. According to the Summary of Product Characteristics (SPC), the maximum recommended treatment duration is 28 days, a limit established from clinical trial data. Courses longer than 14 days require blood count monitoring to detect myelosuppression. In clinical practice, however, treatment of some complex foci can extend over several months, and LZD is an interesting option as it allows oral outpatient therapy [6]. In such cases, the risk of hematological toxicity, particularly thrombocytopenia, can increase [7]. Risk factors include pre-existing renal impairment or high area under the curve (AUC) values [7]. Additional risk factors include concurrent interventions such as renal replacement therapy [8] or the use of P-glycoprotein (P-gp) inhibitors, such as amiodarone, verapamil or certain azole antifungal agents [9].

The clinical relevance of TDM for LZD is supported by large-scale, long-term clinical studies [10, 11] and endorsed by expert panels [1, 12]. TDM has been shown to improve both the safety and efficacy of LZD by addressing its large interindividual variability, especially in high-risk populations such as patients with renal or hepatic impairment, those who are underweight or obese, and critically ill patients [13]. Because LZD and its metabolites share structural features with other myelotoxic drugs, its long-term toxicity profile has raised concern [14].

**Fig. 1:**
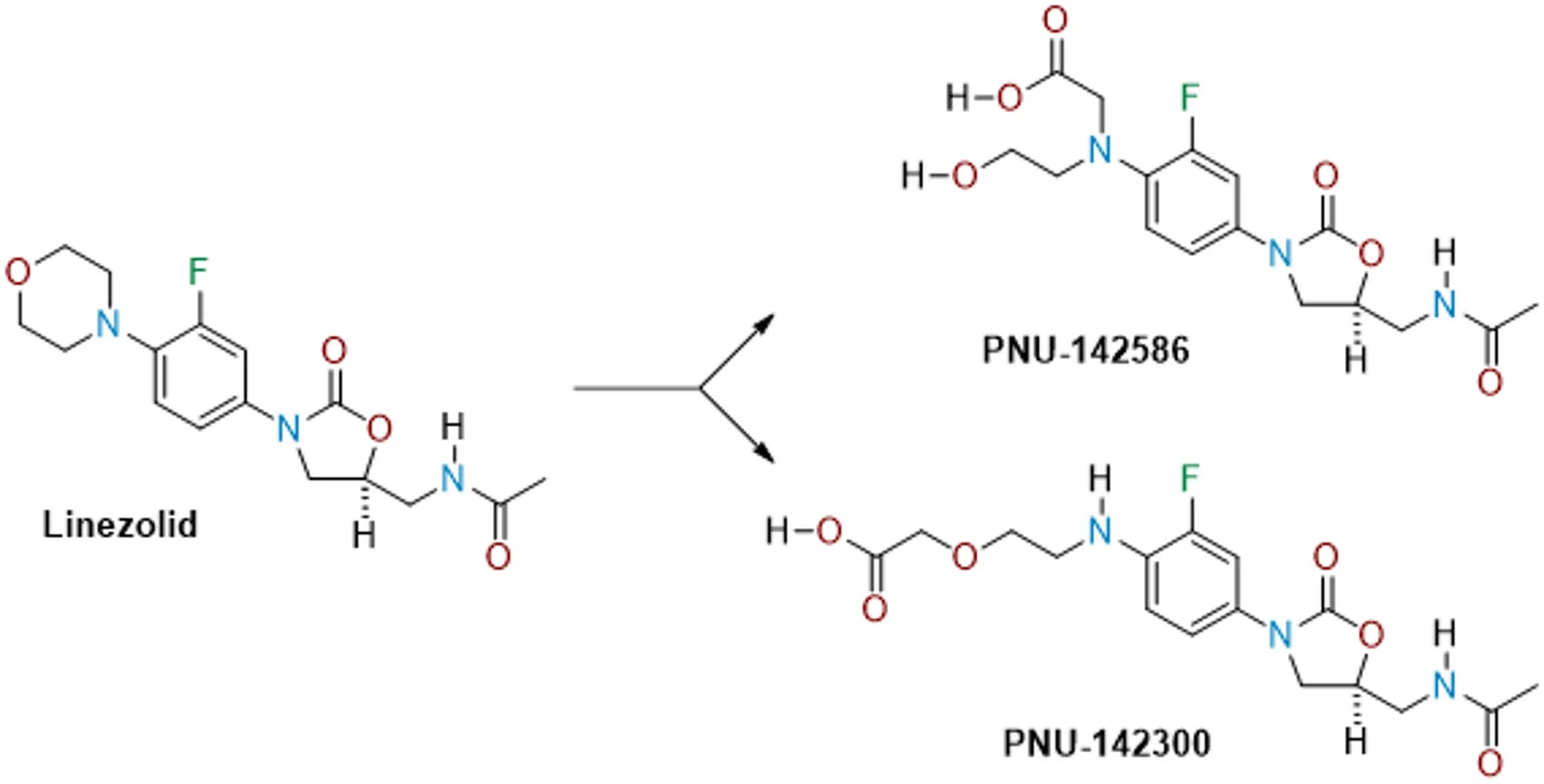
Chemical structures of linezolid and its two main metabolites, PNU-142586 and PNU-142300

Once administered, LZD undergoes metabolic transformation, primarily yielding two main metabolites, PNU-142586 and PNU-142300 (Fig. 1). They are formed by ring-opening of its morpholine moiety through different, not yet fully understood pathways [15]. For years, they were considered pharmacologically inactive. More recent studies, however, suggest that they may contribute to hematological toxicity. For example, PNU-142300 has been shown to predict thrombocytopenia risk more accurately than LZD levels in critically ill patients [16], while PNU-142586 has been linked to direct cytotoxic effects on platelets [17].

Both metabolites accumulate to a greater extent in patients with renal dysfunction. PNU-142586 shows proportionally higher accumulation than PNU-142300 regardless of renal function [14]. One study found that LZD-related adverse effects, such as thrombocytopenia and anemia, correlate with cumulative serum LZD exposure but not with metabolite levels, and reported significantly increased metabolite levels in moderate to severe hepatic and renal impairment. Importantly, metabolite concentrations did not correlate with LZD levels. This highlights the limitations of monitoring the parent drug alone [14]. Pharmacokinetic models that incorporate both metabolites reveal exponential accumulation of LZD and metabolites as renal function declines [18]. Furthermore, the improved safety profile of other oxazolidinones may be due to the absence of a metabolite analogous to PNU-142586 [19].

Despite this growing evidence, current TDM of LZD primarily relies on plasma measurements of the parent drug. Sakurai et al., however, measured plasmatic levels of LZD and its metabolites [20] and developed a pharmacokinetic model incorporating them [18]. Nevertheless, this approach poses practical challenges in certain populations, such as pediatric or geriatric patients, where drawing large volumes of venous blood is often impractical. Less invasive matrices, such as capillary blood or oral fluid (OF), offer promising alternatives.

To enable the use of capillary blood in clinical practice, microsampling techniques have been developed. These techniques use sample volumes below 100 µL and offer several advantages. They are faster, safer (lower contamination and biohazard risk) and more cost-effective, as they do not require specialized personnel or centrifugation prior to storage [21]. Samples also show improved stability, reducing storage and transport demands compared with conventional venous sampling. These features make home-based TDM feasible, allowing patients to collect and mail samples to the laboratory. This approach is particularly valuable for outpatients who require frequent follow-up, including pediatric patients, those with limited mobility or venous access, or individuals with constrained financial resources [22, 23].

Dried blood spot (DBS) sampling, which involves collecting capillary blood via fingertip puncture on filter paper that is then dried and stored, offers a minimally invasive, practical option. DBS enables convenient home collection, reduces the risk of biological contamination, and provides extended sample stability at elevated temperatures [24]. Volumetric absorptive microsampling (VAMS) is an emerging technique that overcomes a common limitation of DBS sampling, hematocrit (Hct)-related variability, by collecting a fixed blood volume regardless of Hct levels [25]. Its potential has already been explored for the quantification of antibiotics, including LZD, in clinical samples [26].

In parallel, OF has emerged as an even less invasive alternative matrix suitable for repeated sampling. It can be easily collected without trained personnel or puncture devices. OF is already a well-established biological matrix for detecting drugs of abuse in forensic and roadside testing contexts and for TDM. Specifically, OF concentrations of LZD have shown strong correlations with plasma concentrations in both healthy volunteers [27] and MDR-TB patients [28, 29]. Furthermore, its applicability has also been explored in preclinical models [30].

To date, and to the best of our knowledge, VAMS-based capillary blood sampling had not previously been clinically validated for linezolid monitoring. Moreover, neither PNU-142300 nor PNU-142586 had been quantified in OF or capillary blood, nor correlated with venous plasma concentrations.

To address these gaps, this study pursued three interrelated objectives. First, to develop and validate a UPLC-MS/MS bioanalytical method for the simultaneous quantification of linezolid, PNU-142300, and PNU-142586 in OF and in capillary blood collected with VAMS, following ICH M10 (EMA, 2022) guidelines. Second, to conduct a clinical evaluation of the validated method using paired samples from patients receiving linezolid therapy, obtained simultaneously across all matrices. And third, to determine whether concentrations measured in these non-invasive matrices correlate sufficiently with venous plasma to support their potential use as alternatives in routine TDM, and to identify the conditions and limitations under which such substitution could be clinically implemented.

## Methods

### Solvents and analytical standards

All solvents were LC-MS grade. Acetonitrile (ACN) and methanol were purchased from Fisher Chemical (Waltham, MA, USA; Refs. A/0638/17 and M/4062/17, respectively), and water was purchased from VWR Chemicals (Radnor, PA, USA; Ref. 83645.320). Analytical standards for LZD (CAS 165800-03-3, Ref. MM3300.00), LZD-d_3_ (CAS 1127120-38-0, Ref. L466502) and PNU-142586 sodium salt (CAS of the free acid: 368891-70-7; Ref. P653405) were obtained from LGC Standards (Wesel, Germany). PNU-142300 (CAS 353263-24-2) was synthesized by Bilarwa Labs (Hyderabad, India).

### Materials and equipment

Certified amber glass LCGC vials (2 mL, 12 × 32 mm) with caps and pre-slit PTFE/silicone septa and low volume (150 µL) inserts with preinstalled plastic springs were both from Waters Corporation (Milford, MA, USA; Refs. 186000847 C and WAT094171, respectively). Glass test tubes were purchased from Labbox (Barcelona, Spain, Ref. TU02-127-250).

In all cases, filtration was performed using 1 mL three-part syringes with concentric Luer tips (CV Médica, Tarragona, Spain; Ref. 8500001). For OF and plasma samples, non-sterile hydrophilic PTFE/L syringe filters (0.2 μm pore size, 13 mm diameter) were used (CHMLab, Barcelona, Spain; Ref. STF020015H/L). For VAMS-derived samples, non-sterile PVDF syringe filters (0.22 μm pore size, 4 mm diameter) were used.

Unless otherwise specified, all centrifugation steps were performed using a model 5804 R centrifuge equipped with a FA-45-48-11 fixed-angle rotor (Eppendorf, Germany).

### Sample origin and ethical approval

Samples used for the development and optimization of the analytical methods included whole blood and plasma obtained from healthy volunteers through a collaboration agreement with the Galician Agency for Blood and Organ Donation (Axencia de Doazón de Órganos e Sangue, ADOS; Santiago de Compostela, Spain) and OF kindly provided by colleagues from the Health Research Institute of Santiago de Compostela (IDIS).

The study was approved by the Galician Network of Ethics Committees (Ethical Approval Code: 2023/048), with authorization recognized across Spain and registration in the Spanish Clinical Studies Registry (Registro Español de Estudios Clínicos, REec), under code 0034-2023-OBS. All procedures were conducted in accordance with Good Clinical Practice guidelines and the ethical principles set forth in the Declaration of Helsinki, as last revised (Helsinki 2024). Written informed consent was obtained from all participants prior to their enrolment in the study.

### Sample collection

OF samples were collected using Salivette^®^ devices (Sarstedt, Nümbrecht, Germany, Ref. 51.1534) following the manufacturer’s instructions. Venous blood was collected in tubes containing ethylenediaminetetraacetic acid (EDTA) as an anticoagulant and centrifuged to obtain plasma. VAMS devices, Mitra™ with 20 µL tips (Neoteryx, Torrance, CA, USA; Ref. 20004), were loaded either directly from capillary blood obtained via finger puncture, following the manufacturer’s instructions, or by dipping their tips into venous blood collected in EDTA-coated tubes. Paired samples were collected simultaneously from each subject.

To improve clarity regarding the different matrices and sampling routes used throughout the study, Table 1 summarizes the role of each specimen type in method validation and clinical comparison. Briefly, plasma obtained from venous blood was used as the reference matrix for clinical comparison. VAMS devices were loaded either with venous whole blood collected in EDTA tubes, to evaluate the performance of the microsampling device independently of capillary sampling, or directly with capillary blood obtained by finger puncture, representing the intended minimally invasive sampling approach. OF was collected using Salivette^®^ devices and evaluated as an alternative non-invasive matrix.

**Table 1:** Summary of matrices, sampling routes and use in the study

Matrix/Specimen type	Sampling route or device	Sample source	Use in the study
Plasma	Venous blood collected in EDTA tubes and centrifuged	Healthy-volunteer samples	Analytical validation
		Patient samples	Reference matrix for clinical comparison
VAMS loaded with venous whole blood (VAMS_WB)	Mitra™ VAMS 20 µL tips loaded by dipping into EDTA venous whole blood	Healthy-volunteer samples	Analytical validation
			Assessment of the VAMS device effect using venous whole blood
		Patient samples	Comparison with plasma and capillary VAMS
VAMS loaded with capillary blood (VAMS)	Mitra™ VAMS 20 µL tips loaded directly after finger puncture	Patient samples	Clinical comparison of the intended minimally invasive capillary sampling approach with plasma and VAMS_WB
Oral fluid (ORAL_FLUID)	Salivette^®^ devices	Healthy-volunteer samples	Analytical validation
		Patient samples	Clinical comparison of this non-invasive alternative matrix with plasma

### Preparation of stock solutions and samples

Stock solutions were prepared as follows: the LZD stock solution at 10 mg/mL in dimethyl sulfoxide (DMSO) was diluted to 1 mg/mL with ACN; PNU-142586 was prepared at 400 µg/mL using an 80:20 (v/v) ACN: water mixture; PNU-142300 was prepared at 2 mg/mL in DMSO and diluted to 1 mg/mL with ACN. The internal standard (IS) LZD-d_3_ stock solution was prepared at 10 mg/mL in DMSO, diluted to 1 mg/mL with ACN, and further diluted to 2 µg/mL with water.

A combined stock solution containing LZD, PNU-142586, and PNU-142300 at 100 µg/mL each was prepared in water and was serially diluted to prepare the working solutions. Two independent sets of working solutions were prepared: one for the calibration curve and another for the quality controls (QCs). Samples were prepared by spiking 10 µL of the corresponding working solution into 190 µL of the matrix of interest (OF, plasma, or whole blood) to obtain calibration points ranging from 0.1 to 5 µg/mL and QCs at 0.2 (low), 2 (medium), and 4 (high) µg/mL.

For samples with controlled Hct levels, whole blood was centrifuged in calibrated tubes, and Hct was adjusted manually by transferring plasma between tubes to achieve final values of 0.25 (low), 0.45 (medium), and 0.70 (high).

### Sample processing

#### VAMS

Procedure was partially adapted from previously published methods [26, 31]. After loading, VAMS were placed to dry at room temperature for a minimum of 2 h, never exceeding a drying time of 24 h. If not processed on the day of collection, VAMS were stored at -20 °C in plastic blisters sealed within plastic freezer bags containing desiccant sachets. For processing, each VAMS tip was pre-hydrated with 50 µL of water followed by vortex mixing. The tips were handled with gloved fingers, which were cleaned with ethanol and dried between samples. Then, 20 µL of a 2 µg/mL IS solution of LZD-d_3_ and 180 µL of methanol were added. The mixture was homogenized in a vortex, sonicated for 15 min in an ultrasonic bath, and centrifuged (12,700 rpm, 10 min, 4 °C). Subsequently, 180 µL of the supernatant were transferred to glass test tubes and evaporated to dryness at 40 °C under a gentle stream of nitrogen. Residues were reconstituted in 100 µL of a 10:90 (v/v) ACN: water mixture. The resulting solutions were transferred into 0.2 mL microtubes (Deltalab, Barcelona, Spain, Ref. 4094.1 N), centrifuged again (5,000 rpm, 10 min, 4 °C), and the supernatants filtered into 150 µL inserts placed in amber LC vials.

#### OF and plasma

Procedures were adapted from previously published methods [20]. OF was recovered from the Salivette^®^ devices by centrifugation (5000 rpm, 10 min, 4 °C) in an Orto Alresa (Madrid, Spain) Digicen 21R centrifuge equipped with an RT 138 angle-fixed rotor. OF and plasma samples were processed in the same way. If not processed on the day of collection, both samples were aliquoted in Eppendorf tubes and stored at -80 °C. Briefly, 50 µL of a 2 µg/mL IS solution of LZD-d_3_ were added to 200 µL of sample, followed by 600 µL of ACN, vortex mixing and centrifugation (12,700 rpm, 10 min, 4 °C). Then, 600 µL of supernatant were transferred to a glass test tube and evaporated to dryness at 40 °C under a nitrogen stream. Residues were reconstituted in 600 µL of a 10:90 (v/v) ACN: water mixture and filtered into amber LC vials.

### UPLC-MS/MS analysis

Detection and quantification of LZD, PNU-142586, and PNU-142300 were carried out using ultra-high-performance liquid chromatography coupled to tandem mass spectrometry (UPLC-MS/MS). The system consisted of an ACQUITY UPLC^®^ H-Class PLUS equipped with a Quaternary Solvent Manager (QSM) and an FTN-H Sample Manager, coupled to a Xevo TQD^®^ triple quadrupole mass spectrometer, controlled through MassLynx V4.2 software. All components, including the software, were from Waters Corporation (Milford, MA, USA).

Chromatographic separation was achieved on an ACQUITY UPLC^®^ BEH C18 column (130 Å, 1.7 μm, 2.1 × 50 mm; Waters™, Ref. 186002350), protected with an ACQUITY UPLC^®^ BEH C18 VanGuard™ pre-column (130 Å, 1.7 μm, 2.1 × 5 mm; Waters™, Ref. 186003975) using a gradient elution with mobile phase A (water with 0.1% formic acid) and mobile phase B (ACN with 0.1% formic acid), at a flow rate of 0.4 mL/min. The elution profile (total run time: 6 min) was as follows: 0–2.0 min, 10% B; 2.0–3.0 min, linear increase to 90% B; 3.0–5.0 min, 90% B; 5.0–5.1 min, linear decrease back to 10% B; and 5.1–6.0 min, 10% B. The column was maintained at 40 °C and the autosampler at 6 °C. The injection volume was 5 µL for OF and VAMS samples and 2 µL for plasma samples.

Analytes were detected in positive electrospray ionization mode (ESI+) using multiple reaction monitoring (MRM). Details of the monitored transitions and MS parameters are provided in the Supporting Information (Table S1).

### Method development and validation

The analytical method was adapted from previously published methods [32, 33] and validated following European Medicines Agency (EMA) ICH M10 guidelines 2022 [34]. Validation parameters included linearity, limit of detection (LOD), limit of quantification (LOQ), accuracy, precision and selectivity. Additional assessments covered matrix effect, extraction efficiency, process efficiency, carryover, dilution integrity, Hct influence, autosampler stability, and storage stability.

Linearity was assessed by preparing 5 calibration curves, each with 6 concentration levels and a zero-concentration level, on 5 different days. Acceptance criteria included a coefficient of determination (r^2^) ≥ 0.975 and residuals within ± 15% (± 20% at lower limit of quantification-(LLOQ)) for at least 75% of calibrators. LOD, defined as the lowest concentration at which both monitored MRM transitions were detectable with a signal-to-noise ratio ≥ 3 and an acceptable ion ratio, was determined by the analysis of three replicates fortified at decreasing concentrations. LOQ, defined as the lowest concentration that could be quantified with adequate precision (%CV ≤ 20%) and accuracy (within ± 20% of the nominal concentration), was determined in triplicate over three different runs using matrix samples from three independent sources.

Accuracy was evaluated by analyzing 5 replicates of low, medium, and high concentration QCs on 3 separate days. Results were expressed as % of the nominal concentration and accepted within 85%–115% of the target value. These data were also used to calculate intraday, interday, and total imprecision, by the calculation of the coefficient of variation (%CV).

For selectivity, 6 blank samples obtained from independent sources were processed and analyzed without fortifying or adding the IS, to verify the absence of endogenous interference at the retention times of the analytes and the IS. For interference studies, separate samples were also fortified with each compound individually at the upper limit of quantification (ULOQ), to evaluate potential cross-interference among analytes. Acceptance required the analyte signal not exceed 10% of the signal measured at the LOQ.

The matrix effect was assessed by analyzing 6 different blank samples from different sources fortifying at the low and high QC levels after extraction, and comparing them to 10 consecutive injections of low and high concentration solutions prepared in mobile phase. The same samples were used to calculate extraction efficiency by comparing blank samples fortifying before and after extraction at both QC levels (*n* = 6). Total process efficiency was determined by comparing peak areas of samples spiked before extraction with those prepared directly in mobile phase.

Carryover was assessed by injecting a blank matrix after the ULOQ daily over five days. It was considered negligible when the signal did not exceed 20% of that of the LOQ for the analytes and 5% for the IS.

Dilution integrity was evaluated by processing samples prepared at four times the ULOQ, diluting them 1:5 and 1:10 prior to analysis (*n* = 5), and comparing the resulting peak areas with those of undiluted samples at the corresponding theoretical concentrations. Results were deemed acceptable when bias was within ± 15% and %CV < 15%.

The effect of the Hct levels was investigated using plasma-corrected blank whole blood samples prepared at three Hct values: 0.25 (low), 0.45 (medium), and 0.70 (high). Each sample was spiked with the analytes of interest at three concentration levels (0.2, 2, and 4 µg/mL) and processed following the standard procedure. The Hct effect was considered acceptable if %CV were ≤ 15%.

To evaluate autosampler stability, low, medium, and high QCs for Salivette^®^ and VAMS samples were processed and analyzed, then kept in the autosampler at 6 °C (operating temperature) and reanalyzed at 72 h. To simulate real storage conditions, 200 µL OF aliquots were stored in Eppendorf tubes at − 80 °C, while VAMS samples were stored at − 20 °C in sealed plastic freezer bags with desiccant sachets. Samples were processed and analyzed at 0 h, 6 weeks, and 3 months.

Unless specified otherwise, conditions were tested in triplicate using independent samples.

### Clinical validation

Paired samples from 35 patients undergoing LZD treatment were analyzed. The full set of samples (VAMS loaded with capillary blood, VAMS loaded with venous whole blood, OF, and plasma) was only available for 21 patients. OF samples could not be collected for 13 of the patients and, for one patient, only plasma and VAMS loaded with venous blood were collected.

### Data processing, analysis, and visualization

Raw data were processed using TargetLynx XS (Waters Corporation, Milford, MA, USA), applying a smoothing algorithm (Mean 2 × 2). All integrations were visually inspected to confirm peak accuracy.

Statistical analyses were conducted in R (version 4.4.3). Data normality was assessed by applying the Shapiro–Wilk test to each variable and to the pairwise differences between methods, complemented by visual inspection of histograms, boxplots, and Q-Q plots (available in the Supporting Information). For each pairwise comparison, Passing-Bablok regression was used to assess agreement, and Pearson’s correlation coefficient to evaluate linear association. Depending on the normality of the differences, either paired t-tests or Wilcoxon signed-rank tests were used to assess statistically significant differences between methods. Additionally, mean percent prediction error (MPPE) and mean absolute percent error (MAPE) were calculated to quantify systematic and overall differences between matrices, and Bland–Altman analysis was performed to visualize bias and limits of agreement. For interpretation of agreement, MPPE and MAPE values within ± 15% and ≤ 15%, respectively, were considered indicative of acceptable agreement between matrices, in line with commonly applied bioanalytical acceptance limits. Values exceeding these thresholds were interpreted as evidence of relevant systematic or overall disagreement, suggesting that matrix-specific correction may be needed before clinical interchangeability can be assumed. Significance was set at *p* < 0.05 in all analyses.

Figures were generated either using GraphPad Prism version 8.0.2 (GraphPad Software, Boston, MA, USA), ChemDraw version 22.2.0 (PerkinElmer, Waltham, MA, USA), or in R using ggplot2 (version 3.5.2).

## Results

### Method validation

A concise overview of the main validation parameters is provided in Table 2. Detailed analyte- and matrix-specific results are described below and further reported in the Supporting Information.

**Table 2:** Validation overview of the UPLC-MS/MS method for LZD, PNU-142586 and PNU-142300 in plasma, oral fluid and VAMS samples

Validation parameter	Summary of results
Calibration range	Linear calibration range from 0.1 to 5 µg/mL for all analytes in plasma, oral fluid and VAMS samples
Accuracy	95.5-110.3% across analytes and matrices
Precision	Within 15% for all analytes and matrices, except for PNU-142586 at the high QC level in VAMS, with total precision of 15.9%
Matrix effect	-24% to 10.9% across analytes and matrices, with acceptable variability (%CV ≤ 8.9%)
Selectivity	No significant endogenous interferences were observed at the retention times of the analytes or internal standard
Carryover	Negligible; blank responses were < 20% of the LLOQ for analytes and < 5% for the internal standard
Autosampler stability	Processed VAMS and oral fluid samples were stable for at least 72 h at 6 °C
Storage stability	Oral fluid samples remained stable for 3 months at − 80 °C. VAMS samples remained stable up to 3 months at − 20 °C, except for PNU-142300, which was stable up to 6 weeks
Dilution integrity	Confirmed for all analytes in plasma, oral fluid, and VAMS samples after 5-fold and 10-fold dilution, with bias within − 11.4% to 8% and precision < 14.9%
Hematocrit influence in VAMS	%CV < 12.1% across most conditions, except for PNU-142586 at the high concentration level, with %CV of 17.2%

The analytical method was validated over a calibration range of 0.1 to 5 µg/mL for LZD, PNU-142300, and PNU-142586 in plasma, OF, and whole blood (capillary or venous) collected with VAMS. Calibration curves were fitted to a linear model using a 1/x² weighting, excluding the origin. Accuracy ranged from 96.4 to 103.1% for VAMS, 99.3-110.3% for OF, and 95.5–109.0% for plasma. Precision, expressed as the coefficient of variation (%CV), was within 15% for all analytes and matrices, except for the total %CV of PNU-142586 at the high QC in VAMS (15.9%). Intra-assay precision ranged from 2.4 to 14% for VAMS, 4.9–14.9% for OF, and 5.9–11.8% for plasma. Inter-assay precision ranged from 0.0 to 9.3% for VAMS, 0.0-3.8% for OF, and 0.0-10.6% for plasma.

For VAMS, matrix effects ranged from − 22% to − 14% for LZD (%CV = 6.5–8.2%), 2% to 6% for PNU-142586 (5.5–8.9%), − 10% to − 1% for PNU-142300 (4.3–5.5%), and − 24% to − 18% for the IS, LZD-d_3_ (5.8–7.6%). In OF, matrix effects varied from − 5.5% to − 3.4% for LZD (1.5–3.1%), 6.0% to 9.4% for PNU-142586 (2.6–4.2%), − 3.5% to 1.0% for PNU-142300 (1.0–5.7%), and − 5.6% for LZD-d_3_ (4.3%). For plasma, matrix effects ranged from − 11.8% to − 10.9% for LZD (%CV = 2.5–2.9%), 8.5% to 10.9% for PNU-142586 (1.9–6.6%), − 2.3% to − 2.1% for PNU-142300 (3.5–3.6%), and − 14.2% to − 10.3% for the IS, LZD-d3 (3.6–4.2%).

No significant interferences were observed at the retention times of the analytes or the IS. Retention times were consistent across matrices: LZD and LZD-d_3_ at approximately 2.10 min, PNU-142586 at 1.80 min, and PNU-142300 at 1.92 min. Carryover was negligible, with blank responses below 20% of the LLOQ for the analytes and 5% for the IS.

**Fig. 2:**
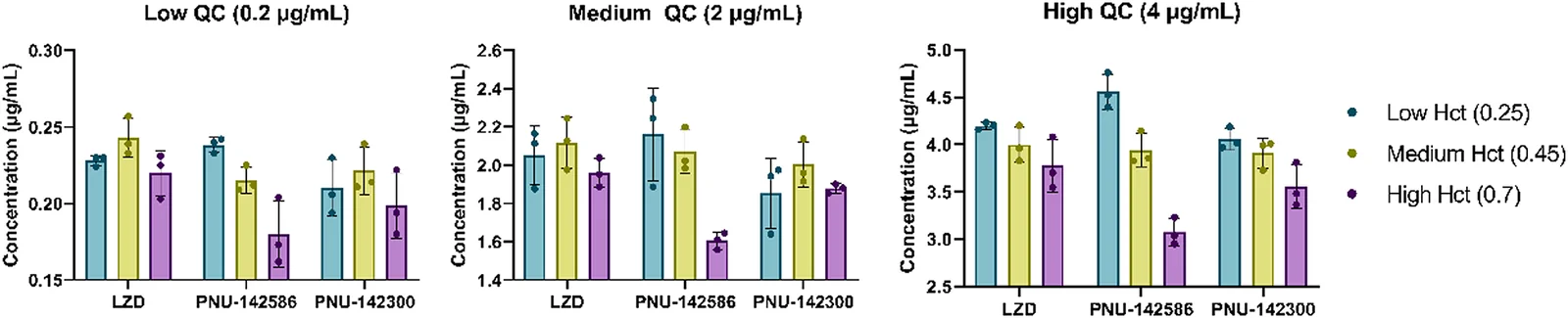
Impact of hematocrit levels on VAMS analyte concentrations. Experimental concentrations of linezolid (LZD), PNU-142586, and PNU-142300 measured in VAMS devices loaded with whole blood (WB) at different hematocrit (Hct) levels (low: 0.25; medium: 0.45; high: 0.70) and analyte concentrations (low: 0.2 µg/mL; medium: 2 µg/mL; high: 4 µg/mL). Each condition was analyzed in triplicate

In VAMS, no significant Hct effect was detected, with %CVs ranging from 0.9 to 7.5% for LZD, 2.0-12.1% for PNU-142586, and 1.3–10.8% for PNU-142300, except for PNU-142586 at high concentrations, where a total %CV of 17.2% was observed (Fig. 2, Table S5).

Processed VAMS and OF samples were found to be stable for at least 72 h when stored at 6 °C in the autosampler, with measured concentrations of all analytes within ± 15% of freshly injected samples.

Samples stored under study conditions were evaluated for stability over 3 months. In VAMS, LZD showed a decrease of − 11.6 to − 13.8% at 3 months, PNU-142586 increased 7.5 to 15.0%, and PNU-142300 ranged from 3.6 to 12.0% at 6 weeks and 14.8 to 25.8% at 3 months. In OF, all analytes remained stable after 3 months (LZD: −1.0 to 1.4%, PNU-142586: −4.4 to 5.1%, and PNU-142300 − 2.7 to 7.9%).

Dilution integrity was verified for all analytes in VAMS, OF and plasma. Processed samples were diluted 5-fold and 10-fold with mobile phase before analysis. Accuracy, expressed as %Bias, ranged from 2.9 to 8% for LZD, − 5.7 to − 8% for PNU-142586, and 0.2 to − 5.0% for PNU-142300 in VAMS; from 1.6 to 2.5%, − 8.2 to − 5.8%, and − 10.1 to − 8.3% in OF; and from − 6.9 to 1.4%, − 9.9 to 4.8%, and − 11.4 to 6.3% in plasma. In all cases, precision (%CV) remained below 15%.

### Analysis of clinical samples

#### Data cleaning and sample pairing

Samples were processed as described above, and all available matrices for each patient were analyzed in the same batch. Suspicious values were excluded from the dataset. Remaining replicates were averaged to a single value per sample, analyte, and matrix, and the resulting values are reported in the Supporting Information (Table S9). After data cleaning, 34 paired samples were initially available for the comparison between plasma and VAMS tips loaded with whole blood (VAMS_WB vs. PLASMA), 31 paired samples for the comparison between VAMS loaded with capillary blood and VAMS loaded with whole blood (VAMS vs. VAMS_WB), 31 paired samples for the comparison between plasma and VAMS loaded with capillary blood (VAMS vs. PLASMA), and 21 paired samples for the comparison between plasma and OF (ORAL_FLUID vs. PLASMA).

#### Descriptive statistics

**Fig. 3:**
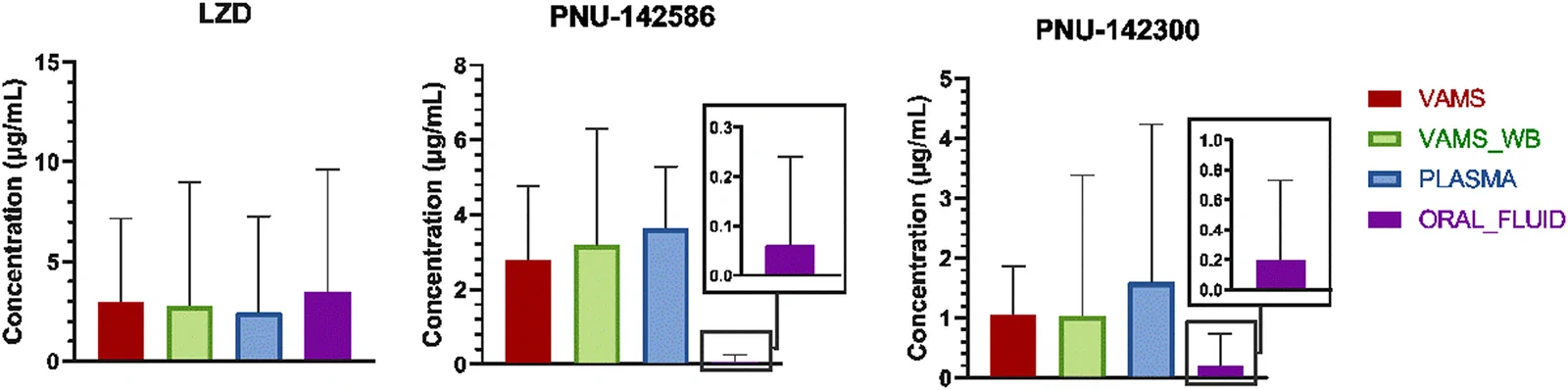
Median concentrations and interquartile ranges of linezolid, PNU-142586 and PNU-142300. Median concentrations (µg/mL) and interquartile ranges of linezolid (LZD) and its metabolites, PNU-142586 and PNU-142300, across the studied matrices. VAMS: volumetric absorptive microsampling devices loaded with capillary blood (*n* = 31); VAMS_WB: VAMS loaded with whole blood (*n* = 35); Plasma (*n* = 34); ORAL_FLUID: Oral fluid collected with Salivette^®^ (*n* = 21)

Concentrations of LZD and its metabolites were measured in VAMS, VAMS loaded with whole blood, plasma, and OF and are summarized in Fig. 3. Descriptive statistics are summarized in the Supporting Information (Table S10 and Figures S7-S15). Median LZD concentrations were broadly similar across matrices, ranging from 2.44 µg/mL in plasma to 3.49 µg/mL in OF. PNU-142586 showed the highest median in plasma (3.65 µg/mL) and the lowest in OF (0.06 µg/mL), with VAMS, either loaded with capillary or venous blood, exhibiting higher maximum concentrations (31.29 and 32.45 µg/mL, respectively) compared with plasma (24.30 µg/mL). For PNU-142300, plasma had the highest median (1.59 µg/mL), while OF had the lowest (0.20 µg/mL). Variability, expressed as standard deviation, was similar across matrices for LZD, generally higher in both VAMS sample types for PNU-142586, and highest in plasma for PNU-142300. All datasets deviated from normality according to the Shapiro–Wilk test (*p* < 0.05).

#### Comparisons across matrices and agreement analyses

A preliminary evaluation was performed to assess the agreement between sampling methods. For each analyte (LZD, PNU-142586, and PNU-142300), VAMS loaded with capillary blood (VAMS), VAMS loaded with whole blood (VAMS_WB), and OF (ORAL_FLUID) were compared with plasma, used as the reference. VAMS was also compared with VAMS_WB.

Passing–Bablok and Pearson’s correlation analyses were performed both including all values (Table S11) and after excluding those below the LOQ (0.1 µg/mL). After applying the LOQ cutoff, Passing-Bablok slopes for VAMS_WB and VAMS compared to plasma were 1.181 and 1.029 (LZD), 0.848 and 0.930 (PNU-142586) and 0.607 and 0.615 (PNU-142300), respectively, with statistically significant (*p* < 0.001) Pearson’s correlations ranging from *r* = 0.899–0.988. Only LZD correlated significantly (*r* = 0.877, *p* < 0.001) between plasma and OF. Slopes for VAMS compared with VAMS_WB ranged from 0.871 to 1.053, with Pearson’s correlations of *r* = 0.917–0.993 (all *p* < 0.001)(Table 3).

**Table 3:** Method comparison results for linezolid (LZD), PNU-142586, and PNU-142300

Analyte	Comparison	n	Test (p-value)	Diff. Sig.?	Passing-Bablok		Pearson’s r (p-value)	Corr. Sig.?
					Intercept (95% CI)	Slope (95% CI)		
LZD	VAMS_WB vs. Plasma	32	Wilcoxon (3.51e-06)	Yes	-0.126 (-0.312 to 0.001)	1.181 (1.122 to 1.267)	0.988 (1.22e-25)	Yes
	VAMS vs. Plasma	28	Wilcoxon (0.150)	No	2.273 (-0.312 to 0.001)	1.029 (0.875 to 1.336)	0.899 (8.14e-11)	Yes
	ORAL_FLUID vs. Plasma	20	Wilcoxon (1.97e-05)	Yes	0.492 (-0.818 to 0.912)	1.143 (1.011–1.713)	0.877 (3.90e-07)	Yes
	VAMS vs. VAMS_WB	29	Wilcoxon (0.468)	No	0.438 (0.009 to 0.771)	0.871 (0.764–1.015)	0.917 (2.83e-12)	Yes
PNU-142586	VAMS_WB vs. Plasma	34	Wilcoxon (0.0781)	No	0.164 (-0.258 to 0.669)	0.848 (0.715–0.959)	0.922 (9.19e-15)	Yes
	VAMS vs. Plasma	30	Wilcoxon (0.017)	Yes	-0.052 (-0.844 to 0.361)	0.930 (0.742 to 1.056)	0.949 (1.61e-15)	Yes
	ORAL_FLUID vs. Plasma	7	Wilcoxon (0.016)	Yes	0.204 (-0.758 to 0.782)	0.016 (-0.059–0.247)	-0.186 (0.689)	No
	VAMS vs. VAMS_WB	31	Paired t (0.226)	No	-0.401 (-0.996 to -0.096)	1.046 (0.904–1.187)	0.982 (1.93e-22)	Yes
PNU-142300	VAMS_WB vs. Plasma	32	Wilcoxon (4.60e-06)	Yes	0.140 (0.046 to 0.236)	0.607 (0.547–0.663)	0.966 (2.92e-19)	Yes
	VAMS vs. Plasma	28	Wilcoxon (6.74e-06)	Yes	0.082 (-0.133 to 0.244)	0.615 (0.492 to 0.743)	0.973 (5.19e-18)	Yes
	ORAL_FLUID vs. Plasma	14	Wilcoxon (2.44e-04)	Yes	0.184 (-0.024 to 0.334)	0.033 (-0.006–0.198)	0.202 (0.489)	No
	VAMS vs. VAMS_WB	29	Wilcoxon (0.275)	No	-0.113 (-0.239 to -0.024)	1.053 (0.960–1.118)	0.993 (8.43e-27)	Yes

Data shown includes sample size (n), statistical difference tests, Passing–Bablok regression parameters (95% CI), and Pearson’s correlation coefficients. VAMS: volumetric absorptive microsampling devices loaded with capillary blood; VAMS_WB: VAMS loaded with whole blood; ORAL_FLUID: Oral fluid collected with Salivette^®^. Values below the LOQ (0.1 µg/mL) were excluded from the analyses

MPPE, MAPE, and MAE were calculated for the main comparisons, both including all data points and excluding pairs below 0.1 µg/mL (Table S12).

Although the between-method differences did not follow a normal distribution, Bland-Altman plots were generated alongside correlation and error analyses for visual inspection (Fig. 5, Table S13). Comparisons were performed for VAMS_WB vs. PLASMA and VAMS vs. VAMS_WB across all analytes, and for ORAL_FLUID vs. PLASMA only for LZD due to the low correlations observed with other analytes.

## Discussion

Our study extends the existing literature by validating VAMS as a non-invasive microsampling strategy for linezolid, incorporating the simultaneous measurement of both major metabolites, and comparing these non-invasive matrices with venous plasma in real patients receiving linezolid therapy. Additionally, OF collected with a Salivette^®^ device is evaluated as an alternative non-invasive matrix, supporting its use for linezolid monitoring while identifying its limitations for metabolite quantification.

During method setup [32, 33], we prioritized unifying and simplifying sample processing as much as possible (e.g., using the same procedure for plasma and saliva). We observed similar matrix effects for LZD and its IS (LZD-d_3_). However, both showed a more pronounced effect in VAMS than in OF. Notably, PNU-142586 exhibited a positive matrix effect in both matrices, whereas PNU-142300 showed minimal matrix influence.

Literature has reported stability of LZD and its metabolites in plasma at − 30 °C for at least one month [20] and of PNU-142300 and LZD in serum for at least three weeks at − 40 °C [32]. They also were found to tolerate freeze–thaw cycles well [20, 32].

Previous studies have evaluated LZD stability in VAMS at − 20 °C for both short- and long-term storage (up to one month) [26]. Consistently, in our study, LZD and its metabolites remained stable for up to six weeks under similar conditions. However, after three months of storage, PNU-142300 showed a slight decrease, observed only in the low and high concentration QCs (25.8 and 20.5%, respectively). The medium QC remained stable (14.8%). This may reflect either minor experimental variability or a modest reduction in metabolite stability. In contrast, LZD and PNU-142586 remained stable throughout the study period.

Another work investigated the short-term stability of LZD in OF. It showed that LZD concentration does not decrease beyond the 15% threshold after incubation for three days at high concentration (20 µg/mL) and temperature (37 °C) [35]. We found that OF samples stored at -80 °C remained stable for at least three months for all analytes. To our knowledge, no previous work has assessed long-term stability of LZD in OF or included its metabolites.

Achieving high extraction yields in VAMS may help minimize Hct-related effects in microsampling. When recovery exceeds approximately 75%, results tend to be less affected even at extreme Hct values. In contrast, lower yields might increase variability due to erythrocyte interference with analyte diffusion into the polymer matrix [25]. In our work, recoveries were generally above this threshold. The only exception was PNU-142586, which showed 75% at the high concentration level. This is consistent with the lower recoveries for this metabolite in plasma reported by Sakurai et al [20].

Regarding Hct effect, mean %CV values were consistently below 15% for all analytes at all concentration levels. The only exception was for PNU-142586, for which the %CV reached 17.2%. This is consistent with the reduced Hct dependence typically observed for VAMS compared to DBS. Studies using DBS have reported underestimation of LZD at Hct values below 45% and overestimation at Hct values above 45% [26]. However, bias remained below 15% for Hct levels between 20% and 50% [24]. Recovery values for LZD in DBS ranged from around 75% using water (to rehydrate) and methanol (extraction solvent) [26] to above 94% with acetonitrile extraction [24].

We initially attempted to validate the analytical method using a PNU-142300 standard from a different supplier. However, we observed major differences in analyte response when comparing spiked blank plasma samples with patient plasma samples. To date, we have not been able to identify the source of these discrepancies, whether due to issues on our side or related to the standard itself.

**Fig. 4:**
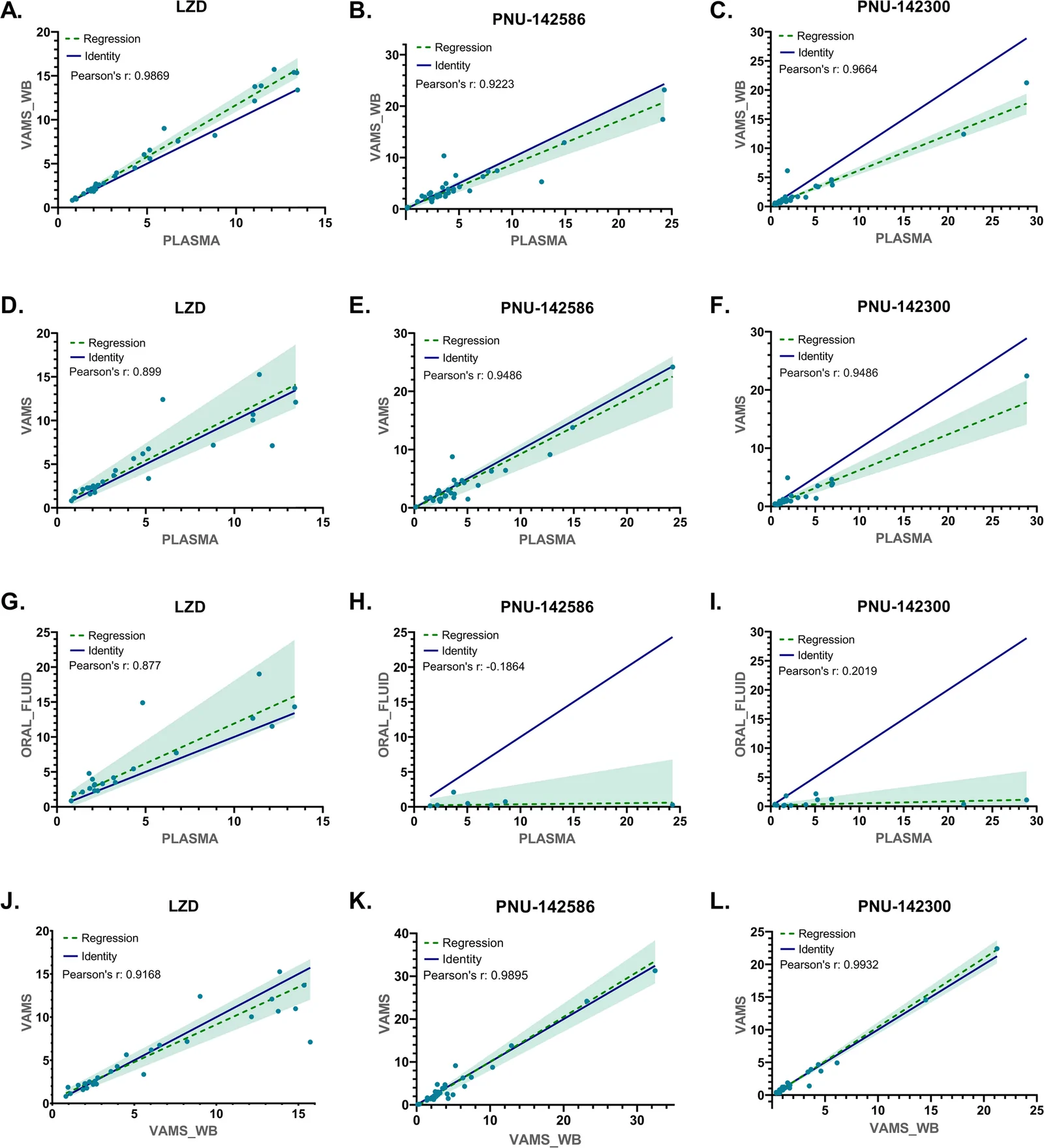
Passing-Bablok regression plots for linezolid (LZD), PNU-142586 and PNU-142300. Panels are organized by metabolite (columns) and comparison (rows): **A-C**, VAMS_WB vs. plasma; **D–F**, VAMS vs. plasma; **G–I**, oral fluid vs. plasma; **J–L**, VAMS vs. VAMS_WB. In all plots, the method defined as reference is shown on the x-axis. VAMS: volumetric absorptive microsampling devices loaded with capillary blood; VAMS_WB: VAMS loaded with whole blood; ORAL_FLUID: Oral fluid collected with Salivette^®^. Shaded areas correspond to the 95% confidence intervals. Values below the LOQ (0.1 µg/mL) were excluded from the analyses

**Fig. 5:**
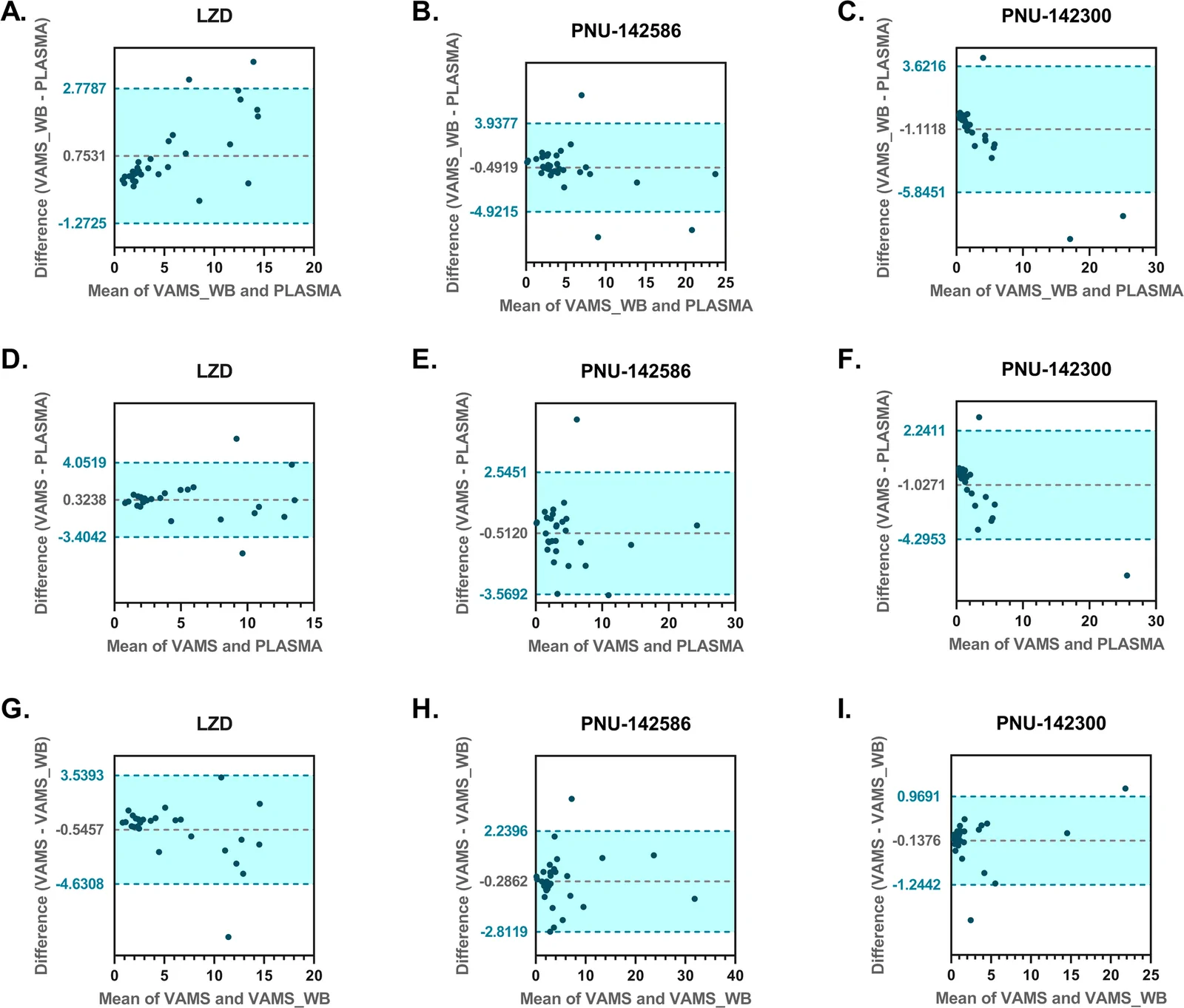
Bland-Altman plots for linezolid (LZD), PNU-142586 and PNU-142300. Panels are organized by metabolite (columns) and comparison (rows): **A-C**, VAMS_WB vs. plasma; **D–F**, VAMS vs. plasma; **G–I**, VAMS vs. VAMS_WB. VAMS: volumetric absorptive microsampling devices loaded with capillary blood; VAMS_WB: VAMS loaded with whole blood; ORAL_FLUID: Oral fluid collected with Salivette^®^. Dashed gray line indicates the mean difference between methods and shaded areas the limits of agreement (mean ± 1.96*SD). Values below the LOQ (0.1 µg/mL) were excluded from the analyses

Plasma was used as the reference matrix and was first compared with VAMS loaded with whole blood (VAMS_WB) (Figs. 4 and 5). This approach is commonly used in the literature to evaluate the impact of the VAMS device on measured analyte concentrations [31]. We found that VAMS slightly overestimated LZD plasma concentrations. However, the correlation between both matrices was strong and significant (*r* = 0.988, *p* < 0.001), with MPPE and MAPE values below the predefined 15% agreement threshold. These results suggest that, for LZD, the methods could be considered largely interchangeable. However, this should be interpreted with caution, as further studies with larger sample sizes are needed to confirm these findings. For both metabolites, correlations with plasma were also strong and significant (*r* = 0.922–0.966, *p* < 0.001). However, VAMS_WB tended to underestimate their concentrations compared to plasma. This bias was particularly evident for PNU-142300, the Passing-Bablok slopes for VAMS_WB vs. plasma and capillary VAMS vs. plasma were 0.607 and 0.615, respectively, suggesting an approximate 38–39% underestimation of plasma concentrations when using VAMS-based measurements. In these cases, both MPPE and MAPE exceeded the predefined 15% agreement threshold, indicating that matrix-specific correction factors should be considered if VAMS were to be used for metabolite quantification. However, these estimates should be interpreted as an indication of the expected magnitude of bias rather than as validated correction factors for clinical use. The present dataset does not support the immediate implementation of fixed proportional correction factors; instead, future studies should derive and externally validate matrix- and analyte-specific conversion equations using venous plasma as the reference matrix. If clinically relevant covariates are shown to influence matrix-to-plasma relationships, more advanced approaches, such as covariate-adjusted regression or population pharmacokinetic models, may be required. These results build on earlier observations of LZD on a smaller dataset [26]. They also extend previous work by including the measurement of its metabolites.

When plasma was compared with VAMS loaded with capillary blood, agreement remained high for all analytes. For LZD, correlation with plasma was strong (*r* = 0.899, *p* < 0.001), with a Passing–Bablok slope close to unity (1.029; 95% CI: 0.875–1.336). For both metabolites, correlation with plasma was even stronger (*r* = 0.949 for PNU-142586 and *r* = 0.973 for PNU-142300). VAMS tended to underestimate concentrations relative to plasma to a similar extent as VAMS_WB. However, the confidence intervals were wider in the VAMS vs. plasma comparison, indicating greater variability.

Next, we compared capillary VAMS to VAMS_WB to evaluate the additional impact of capillary sampling. For LZD, VAMS still tended to slightly underestimate VAMS_WB. For both metabolites, capillary VAMS slightly overestimated concentrations. However, correlations were strong and significant (*p* < 0.001), with slopes close to unity.

The comparison between plasma and OF revealed a more heterogeneous picture. LZD concentrations in OF showed a strong and statistically significant correlation with plasma (*r* = 0.877, *p* < 0.001) (Fig. 4G-I). This indicates that OF correlates relatively well with systemic LZD exposure. These findings align with previous studies reporting high agreement between plasma and OF levels of LZD. They also closely match the correlation observed after intravenous administration (*r* = 0.885) [36], the main route used in our study. LZD concentrations in OF have been reported to exceed plasma levels by roughly 25% [37]. In our dataset, LZD concentrations in OF were on average approximately 50% higher than in plasma. However, patients were at varying treatment durations. The potential influence of timing since administration on OF/plasma ratios was beyond the scope of this study. High correlations between serum and OF have also been reported for LZD (*r* = 0.95) [28]. In contrast, the metabolites were virtually undetectable in OF in most cases. This suggests that this matrix may not be suitable for their quantification, at least until the underlying reasons are further investigated.

The virtual absence of PNU-142300 and PNU-142586 in OF likely reflects several unfavourable physicochemical properties compared to the parent drug. Both metabolites are ring-opened carboxylic acid derivatives with a free carboxylic acid group [38]. Their acidic character increases polarity and promotes ionisation at physiological pH, thereby impeding passive non-ionic diffusion across the salivary gland epithelium, which is the predominant mechanism of drug transfer into OF [39, 40]. Finally, linezolid has a relatively low plasma protein binding of approximately 31%, which facilitates its diffusion into OF [41]. By contrast, the protein binding of the metabolites has not been formally characterised, but the introduction of a free carboxylic acid group is generally associated with increased albumin affinity in small-molecule metabolites, which would further reduce the free fraction available for passive transfer [42].

The comparison between VAMS_WB and plasma showed consistent MPPE, MAPE, and MAE values for LZD when including all data points or after excluding those < 0.1 µg/mL. In contrast, the agreement between VAMS and plasma, as well as between VAMS and VAMS_WB, was markedly distorted when all data points were considered (MPPEs and MAPEs exceeding 200%). This likely reflects artifacts from measurements close to zero. Agreement improved substantially after exclusion of values below 0.1 µg/mL. For PNU-142586 and PNU-142300, data filtering had minimal effect on the overall agreement metrics. These parameters were not calculated for the comparison between OF and plasma due to poor correlations. Thus, the poorer agreement observed when all data points were included was mainly driven by very low concentrations close to the analytical limit. From a clinical perspective, this limitation is unlikely to substantially affect therapeutic decision-making, which generally focuses on higher LZD concentrations within or near the therapeutic range.

Two broad clinical contexts illustrate how these matrices could be integrated into existing TDM workflows. The first encompasses patients receiving prolonged linezolid therapy in ambulatory settings, where repeated venous access is burdensome, toxicity risk accumulates over time, and dose adjustments based on drug exposure could prevent early discontinuation. This is particularly relevant in elderly or frail patients, in whom difficult venous access is common and repeated hospital visits for blood sampling represent a significant logistical and clinical burden. In this context, a VAMS-based workflow could be implemented through patient self-collection via fingertip capillary puncture at a trough time point, followed by drying at room temperature and mail-in submission to the hospital laboratory in a standard envelope with a desiccant sachet. This procedure is shown to be feasible and well accepted by patients in other TDM contexts [21]. The second encompasses patients in resource-limited or decentralised care settings, where venous access and laboratory infrastructure are often constrained. Here, OF collected with a Salivette^®^ device represents a complementary alternative requiring no puncture device, no trained personnel, and minimal patient instruction, and is already established as a feasible matrix for linezolid monitoring in this type of setting [28]. Across both contexts, the laboratory would apply the validated UPLC-MS/MS method described here, use matrix-specific correction factors as needed, and communicate results and dose recommendations remotely to the prescribing clinician. The critical prerequisite for clinical implementation is the prospective definition of matrix-specific therapeutic targets or the systematic application of validated correction factors.

This study has some limitations that should be acknowledged. The clinical validation was performed in a modest sample of 35 patients and in a single-center setting, which may limit the generalizability of the findings. In addition, the study was focused on analytical and matrix-comparison validation and was not designed to directly assess clinical outcomes, such as efficacy or LZD-related toxicity. Further multicenter studies with larger cohorts and clinical outcome data will be needed to confirm these results and assess their clinical relevance.

## Conclusions

In this study, we developed and validated a UPLC-MS/MS method for the simultaneous quantification of LZD and its major metabolites, PNU-142300 and PNU-142586, in non-invasive matrices, specifically OF and capillary blood collected by VAMS. Our findings demonstrate that while correlations between plasma and VAMS were strong for LZD and both metabolites, observed systematic differences indicate that matrix- and analyte-specific correction strategies may be required before routine clinical implementation. In the case of OF, only LZD showed a significant correlation with plasma, limiting the current utility of this matrix for metabolite monitoring.

Overall, these results support the feasibility of microsampling techniques as practical alternatives to conventional venous sampling, particularly in settings where less invasive procedures are desirable. Implementation of this approach could broaden the applicability of TDM for LZD and improve its accessibility. However, further studies in larger cohorts are needed to externally validate matrix- and analyte-specific conversion equations and to determine whether simple proportional correction factors are sufficient or whether covariate-adjusted approaches are required.

## Supplementary Information

Below is the link to the electronic supplementary material.


Supplementary Material 1


## Data Availability

All data generated or analysed during this study are included in this published article and its supplementary information files.

## Abbreviations

ACN: Acetonitrile
ADOS: Axencia de Doazón de Órganos e Sangue
AUC: Area under the curve
CEIm-G: Comité de Ética da Investigación con Medicamentos de Galicia
CI: Confidence interval
CV: Coefficient of variation
DBS: Dried blood spot
DMSO: Dimethyl sulfoxide
EDTA: Ethylenediaminetetraacetic acid
EMA: European Medicines Agency
ESI+: Positive electrospray ionization
Hct: Hematocrit
IDIS: Health Research Institute of Santiago de Compostela
IS: Internal standard
LLOQ: Lower limit of quantification
LOD: Limit of detection
LOQ: Limit of quantification
LZD: Linezolid
MAE: Mean absolute error
MAPE: Mean absolute percent error
MDR-TB: Multidrug-resistant tuberculosis
MPPE: Mean percent prediction error
MRM: Multiple reaction monitoring
MRSA: Methicillin-resistant *Staphylococcus aureus*
OF: Oral fluid
P-gp: P-glycoprotein
PK: Pharmacokinetic
PTC: Peptidyl transferase center
QC: Quality control
QSM: Quaternary Solvent Manager
REec: Registro Español de Estudios Clínicos
SPC: Summary of Product Characteristics
TDM: Therapeutic drug monitoring
ULOQ: Upper limit of quantification
UPLC-MS/MS: Ultra-performance liquid chromatography - tandem mass spectrometry
VAMS: Volumetric absorptive microsampling
VRE: Vancomycin-resistant *Enterococcus*
WHO: World Health Organization
