# Supplementary material for: Simultaneous quantification of linezolid and its metabolites (PNU-142300 and PNU-142586) in oral fluid and capillary blood by UPLC-MS/MS: method validation and clinical application using non-invasive sampling techniques
Source: J Transl Med. 2026 Jun 17;24:1108. doi: 10.1186/s12967-026-08465-8 (PMC13523367; doi:10.1186/s12967-026-08465-8)
Supplement: Supplementary file 1 [file 12967_2026_8465_MOESM1_ESM.docx]

***Supporting information for***

**Simultaneous quantification of linezolid and its metabolites (PNU-142300 and PNU-142586) in oral fluid and capillary blood by UPLC-MS/MS: method validation and clinical application using non-invasive sampling techniques**

Contents

**Table S1.**	MRM transitions and settings for Linezolid (LZD), Linezolid-d3 (LZD-d3), PNU-142586, and PNU-142300.	3

**Table S2.**	Summary of the procedures, acceptance criteria, and results of method validation. LZD: Linezolid; IS: Internal Standard (LZD-d_3_); ULOQ: Upper Limit of Quantification; LLOQ: Lower Limit of Quantification; QC: Quality Control; OF: Oral Fluid; ME: Matrix Effect; PE: Process Efficiency; EE: Extraction Efficiency. * Medium concentration QCs excluded due to experimental error.	4

**Table S3.**	Summary of calibration curve parameters in VAMS for Linezolid (LZD), PNU-142586, and PNU-142300. Curves were prepared on five separate days by multiple operators, each with six calibration levels and a zero level. For each analyte, the table shows the nominal concentrations, the coefficient of determination (r²), the slope and intercept of the linear regression, and the mean, standard deviation (SD), coefficient of variation (%CV), and mean relative error (%MRE) across days. Data fitted to a linear model (1/x² weighting, origin excluded).	6

**Table S4.**	Summary of calibration curve parameters in oral fluid for Linezolid (LZD), PNU-142586, and PNU-142300. Curves were prepared on five separate days by multiple operators, each with six calibration levels and a zero level. For each analyte, the table shows the nominal concentrations, the coefficient of determination (r²), the slope and intercept of the linear regression, and the mean, standard deviation (SD), coefficient of variation (%CV), and mean relative error (%MRE) across days. Data fitted to a linear model (1/x² weighting, origin excluded).	7

**Table S5.**	Summary of calibration curve parameters in plasma for Linezolid (LZD), PNU-142586, and PNU-142300. Curves were prepared on five separate days by multiple operators, each with six calibration levels and a zero level. For each analyte, the table shows the nominal concentrations, the coefficient of determination (r²), the slope and intercept of the linear regression, and the mean, standard deviation (SD), coefficient of variation (%CV), and mean relative error (%MRE) across days. Data fitted to a linear model (1/x² weighting, origin excluded).	8

**Table S6.**	Autosampler stability of Linezolid (LZD), PNU-142586, and PNU-142300 in processed samples after 72 h at 6ºC. Results are expressed as mean percentage difference (%Difference) from freshly injected QC samples (n=3 for each concentration level).	12

**Table S7.**	Experimental concentrations (mean ± SD, n = 3) of Linezolid (LZD), PNU-142586, and PNU-142300 measured in VAMS devices loaded with whole blood at three hematocrit (Hct) levels (Low: 0.25; Medium: 0.45; High: 0.7) and three nominal concentration levels (0.2, 2, and 4 µg/mL).	12

**Table S8.**	Influence of hematocrit (Hct) levels (Low: 0.25; Medium: 0.45; High: 0.7) on assay precision expressed as coefficient of variation (CV%) for Linezolid (LZD), PNU-142586, and PNU-142300 at low, medium, and high concentration levels in VAMS samples.	13

**Table S9.**	Individual linezolid (LZD), PNU-142586, and PNU-142300 concentrations measured in plasma, VAMS loaded with venous whole blood (VAMS_WB), capillary VAMS, and oral fluid (OF). Each row corresponds to a paired sample obtained from the same patient. Suspected outliers were excluded before averaging replicates to yield a single value per sample, analyte, and matrix.	14

**Table S10.**	Median concentrations [interquartile range, IQR], minimum–maximum values, and standard deviations (SD) of linezolid (LZD) and its metabolites PNU-142586 and PNU-142300 in VAMS loaded with capillary blood (VAMS, n = 31), VAMS loaded with whole blood (VAMS_WB, n = 35), plasma (n = 34), and oral fluid collected with Salivette® (ORAL_FLUID, n = 21). All concentrations are expressed in µg/mL.	15

**Table S11.**	Method comparison results for Linezolid (LZD), PNU-142586, and PNU-142300, showing sample size (n), statistical difference tests, Passing–Bablok regression parameters (95% CI), and Pearson’s correlation coefficients. VAMS: volumetric absorptive microsampling devices loaded with capillary blood; VAMS_WB: VAMS loaded with whole blood; ORAL_FLUID: Oral fluid collected with Salivette®. All experimental values were included in the calculation.	19

**Table S12.**	Mean percentage prediction error (MPPE), mean absolute percentage error (MAPE), mean absolute error (MAE), and number of paired samples (n) obtained for each analyte and comparison. VAMS: volumetric absorptive microsampling devices loaded with capillary blood; VAMS_WB: VAMS loaded with whole blood.	19

**Table S13.**	Bland-Altman comparison results. VAMS: volumetric absorptive microsampling devices loaded with capillary blood; VAMS_WB: VAMS loaded with whole blood.	20

***Table S1.***	*MRM transitions and settings for Linezolid (LZD), Linezolid-d3 (LZD-d3), PNU-142586, and PNU-142300.*

|  | **Precursor (m/z)** | **Product (m/z)** | **Cone (V)** | **Collision Energy (eV)** | **Dwell (s)** |
| --- | --- | --- | --- | --- | --- |
| ***LZD*** | 338.05 | 55.93 | 52 | 33 | 0.011 |
|  |  | 295.98 |  | 18 |  |
| ***LZD-d3*** | 341.14 | 56.85 | 50 | 38 | 0.011 |
|  |  | 195.08 |  | 22 |  |
|  |  | 297.30 |  | 22 |  |
| ***PNU-142586*** | 370.16 | 55.94 | 42 | 44 | 0.011 |
|  |  | 148.04 |  | 48 |  |
|  |  | 177.00 |  | 38 |  |
|  |  | 282.03 |  | 24 |  |
| ***PNU-142300*** | 371.78 | 55.94 | 56 | 44 | 0.011 |
|  |  | 136.20 |  | 36 |  |
|  |  | 251.97 |  | 14 |  |

***Table S2.***	*Summary of the procedures, acceptance criteria, and results of method validation. LZD: Linezolid; IS: Internal Standard (LZD-d_3_); ULOQ: Upper Limit of Quantification; LLOQ: Lower Limit of Quantification; QC: Quality Control; OF: Oral Fluid; ME: Matrix Effect; PE: Process Efficiency; EE: Extraction Efficiency. * Medium concentration QCs excluded due to experimental error.*

| **Parameter** | **Procedure** | **Acceptance criteria** | **Matrix** | **Results** |
| --- | --- | --- | --- | --- |
| Linearity and range | 5 calibration curves prepared over 5 different days by multiple operators, each including 6 calibration levels and a zero level, all levels doped with the IS | Mean r² ≥ 0.975; residuals within ±15% (±20% at LLOQ) for ≥75% of calibrators | All matrices | Data fitted to a linear model (1/x² weighting, origin excluded). Linear range: 0.1-5 µg/mL for all analytes and matrices. See Table S3 (VAMS),Table S4 (OF), and Table S5 (Plasma) |
| Accuracy | 5 replicates at the low, medium, and high QC level in 3 different days (n=15) | 85–115% of nominal concentration | VAMS | LZD: 98.5-97.8%; PNU-142586: 96.4-99.6%; PNU-142300: 98.1-103.1% |
|  |  |  | OF | LZD: 99.3-110.3%; PNU-142586: 102.1-108.6%; PNU-142300: 102.3-108.9% |
|  |  |  | Plasma | LZD: 95.8-107.8%; PNU-142586: 95.5-101.2%; PNU-142300: 97.4-109.0% |
| Precision | 5 replicates at the low, medium, and high QC level in 3 different days (n=15) | %CV <15% | VAMS | Intra-assay – LZD: 2.4–10.6%, PNU-142586: 10.0–13.0%, PNU-142300: 10.3–14.0%  Inter-assay – LZD: 0.0–9.3%, PNU-142586: 6.4–9.1%, PNU-142300: 0.0–3.8%  Total imprecision – LZD: 9.6–10.8%, PNU-142586: 11.9–15.9%, PNU-142300: 10.8–14.0% |
|  |  |  | OF | Intra-assay – LZD: 5.4–14.9%, PNU-142586: 5.0–13.8%, PNU-142300: 4.9–12.1%  Inter-assay – LZD: 0.0–2.1%, PNU-142586: 0.0–3.8%, PNU-142300: 0.0–3.7%  Total imprecision – LZD: 5.4–15.0%, PNU-142586: 6.2–14.3%, PNU-142300: 6.2–12.1% |
|  |  |  | Plasma | Intra-assay – LZD: 5.9–8.0%, PNU-142586: 7.7–11.8%, PNU-142300: 6.8–9.8%  Inter-assay – LZD: 0.0–1.5%, PNU-142586: 0.0–5.3%, PNU-142300: 0.0–10.6%  Total imprecision – LZD: 5.9-8.0%, PNU-142586: 8.6-11.8%, PNU-142300: 8.1-14.5% |
| Selectivity | Blank matrices from ≥6 donors. Analytes spiked individually at the ULOQ | Interference ≤20% of LLOQ analyte, ≤5% IS; no significant signal at analyte or IS Rt | All matrices | No significant interference observed |
| Carry-over | Blank samples injected immediately after ULOQ calibrator (n=5) | Analyte signal ≤20% of LLOQ; IS signal ≤5% | All matrices | No carry-over found |
| Matrix effect | Post-extraction spiked samples from independent sources (n=6) versus neat standards at low and high QC levels (n=10) | %CV ≤ 15% | VAMS | ME (%CV): LZD: -22 to −14% (6.5-8.2%); PNU-142586: 2 to 6% (5.5-8.9%); PNU-142300: −10 to −1% (4.3-5.5%); LZD-d3: −24 to −18% (5.8-7.6%) |
|  |  |  | OF | ME (%CV): LZD: −5.5 to −3.4% (1.5–3.1%); PNU-142586: 6.0 to 9.4% (2.6–4.2%);  PNU-142300: −3.5 to 1.0% (1.0–5.7%); LZD-d3: −5.6% (4.3%) |
|  |  |  | Plasma | ME (%CV): LZD: −11.8 to −10.9% (2.5–2.9%); PNU-142586: 8.5 to 10.9% (1.9–6.6%);  PNU-142300: −2.3 to −2.1% (3.5–3.6%); LZD-d3: −14.2 to −10.3% (3.6–4.2%) |
| Autosampler stability | Processed samples at the low, medium, and high QC level: injected, stored in the autosampler at 6°C, and re-injected after 72 h (n=3) | Mean concentration within ±15% of freshly prepared samples | VAMS | All analytes were stable (%loss: -5.2 to 3.8%) |
|  |  |  | OF | All analytes were stable (%loss: -0.1 to 5.4%) |
|  |  |  | Plasma | Not assessed |
| Storage stability | Samples at the low, medium, and high QC level stored at –20 °C in sealed plastic bags with silica gel for VAMS, and at –80 °C in Eppendorf tubes for OF; processed and analyzed at t=0, 6 weeks, and 3 months (n=3) | Mean concentration within ±15% of freshly prepared samples | VAMS | LZD (-11.6 to -13.8%) at 3 months; PNU-142586 (7.5 to 15.0% at 3 months); PNU-142300 (3.6 to 12.0% at 6 weeks, 14.8 to 25.8% at 3 months) |
|  |  |  | OF* | LZD (-1.0 to 1.4%) at 3 months; PNU-142586 (-4.4 to 5.1% at 3 months); PNU-142300  (-2.7 to 7.9% at 3 months) |
|  |  |  | Plasma | Not assessed |
| Dilution integrity | Samples with concentrations at 4×ULOQ were processed, then diluted 1:5 and 1:10 before analysis (n=5) | %Bias within ±15%, %CV <15% | VAMS | %Bias (%CV): LZD: 2.9 to 8% (1.5-1.7%), PNU-142586: -5.7 to -8% (2.5-3.5%), PNU-142300: 0.2 to -5.0% (1.6-3.1%) |
|  |  |  | OF | %Bias (%CV): LZD: 1.6 to 2.5% (2.1–11.2%), PNU-142586: –8.2 to –5.8% (4.3–13.9%), PNU-142300: –10.1 to –8.3% (4.6–13.8%) |
|  |  |  | Plasma | %Bias (%CV): LZD: –6.9 to 1.4% (12.2–12.8%), PNU-142586: –9.9 to 4.8% (13.3–14.2%), PNU-142300: –11.4 to 6.3% (13.0–14.9%) |

***Table S3.***	*Summary of calibration curve parameters in VAMS for Linezolid (LZD), PNU-142586, and PNU-142300. Curves were prepared on five separate days by multiple operators, each with six calibration levels and a zero level. For each analyte, the table shows the nominal concentrations, the coefficient of determination (r²), the slope and intercept of the linear regression, and the mean, standard deviation (SD), coefficient of variation (%CV), and mean relative error (%MRE) across days. Data fitted to a linear model (1/x² weighting, origin excluded).*

| ***Analyte:*** | ***LZD*** |  |  |  |  |  | |  |  |  |
| --- | --- | --- | --- | --- | --- | --- | --- | --- | --- | --- |
| **Nominal conc.** | ***Curve #1*** | ***Curve #2*** | ***Curve #3*** | ***Curve #4*** | ***Curve #5*** | **Mean** | **SD** | | **%CV** | **%MRE** |
| ***0*** | 0 | 0 | 0.026 | 0.002 | 0.012 | 0.008 | 0.01 | |  |  |
| ***0.1*** | 0.106 | 0.1 | 0.102 | 0.094 | 0.104 | 0.101 | 0.00 | | 4.5 | -1.20 |
| ***0.25*** | 0.231 | 0.249 | 0.252 | 0.292 | 0.24 | 0.253 | 0.02 | | 9.3 | -1.12 |
| ***0.5*** | 0.416 | 0.515 | 0.45 | 0.483 | 0.448 | 0.462 | 0.04 | | 8.2 | 7.52 |
| ***1*** | 1.037 | 0.996 | 0.952 | 1.079 | 0.944 | 1.002 | 0.06 | | 5.7 | -0,16 |
| ***2.5*** | 2.563 | 2.663 | 2.567 | 2.264 | 2.517 | 2.515 | 0.15 | | 6.0 | -0.59 |
| ***5*** | 5.633 | 4.594 | 5.472 | 4.718 | 5.776 | 5.239 | 0.54 | | 10.4 | -4.77 |
| ***r^2^*** | 0.984 | 0.997 | 0.994 | 0.985 | 0.988 | **0.989** | **0.01** | | 0.6 |  |
| ***Intercept*** | 0.895 | 0.244 | 0.199 | 0.0244 | 0.034 | **0.279** | **0.36** | | 128.1 |  |
| ***Slope*** | 19.036 | 18.397 | 23.952 | 22.374 | 18.745 | **20.501** | **2.50** | | 12.2 |  |
|  |  |  |  |  |  |  |  | |  |  |
| ***Analyte:*** | ***PNU-142586*** | |  |  |  |  |  | |  |  |
| **Nominal conc.** | ***Curve #1*** | ***Curve #2*** | ***Curve #3*** | ***Curve #4*** | ***Curve #5*** | **Mean** | **SD** | | **%CV** | **%MRE** |
| ***0*** | 0 | 0 | 0.034 | 0.011 | 0.01 | 0.011 | 0.01 | |  |  |
| ***0.1*** | 0.107 | 0.097 | 0.104 | 0.094 | 0.105 | 0.101 | 0.01 | | 5.5 | -1.40 |
| ***0.25*** | 0.232 | 0.273 | 0.238 | 0.288 | 0.25 | 0.256 | 0.02 | | 9.3 | -2.48 |
| ***0.5*** | 0.403 | 0.479 | 0.46 | 0.509 | 0.402 | 0.451 | 0.05 | | 10.5 | 9.88 |
| ***1*** | 0.946 | 1.024 | 0.952 | 1.054 | 0.896 | 0.974 | 0.06 | | 6.5 | 2.56 |
| ***2.5*** | 2.395 | 2.548 | 2.454 | 2.198 | 2.439 | 2.407 | 0.13 | | 5.4 | 3.73 |
| ***5*** | 6.464 | 4.691 | 5.784 | 4.811 | 6.399 | 5.630 | 0.85 | | 15.0 | -12.60 |
| ***r^2^*** | 0.962 | 0.996 | 0.989 | 0.987 | 0.964 | **0.979** | **0.02** | | 1.6 |  |
| ***Intercept*** | 0.076 | 0.053 | 0.007 | -0.036 | 0.007 | **0.021** | **0.04** | | 207.6 |  |
| ***Slope*** | 2.904 | 2.983 | 3.388 | 3.359 | 3.066 | **3.140** | **0.22** | | 7.0 |  |
|  |  |  |  |  |  |  |  | |  |  |
| ***Analyte:*** | ***PNU-142300*** | |  |  |  |  |  | |  |  |
| **Nominal Conc.** | ***Curve #1*** | ***Curve #2*** | ***Curve #3*** | ***Curve #4*** | ***Curve #5*** | **Mean** | **SD** | | **%CV** | **%MRE** |
| ***0*** | 0 | 0 | 0.032 | 0.007 | 0.007 | 0.009 | 0.01 | |  |  |
| ***0.1*** | 0.108 | 0.1 | 0.104 | 0.095 | 0.109 | 0.103 | 0.01 | | 5.6 | -3.20 |
| ***0.25*** | 0.226 | 0.253 | 0.242 | 0.273 | 0.215 | 0.242 | 0.02 | | 9.4 | 3.28 |
| ***0.5*** | 0.376 | 0.485 | 0.439 | 0.518 | 0.418 | 0.447 | 0.06 | | 12.5 | 10.56 |
| ***1*** | 1.007 | 1.043 | 0.949 | 1.094 | 0.92 | 1.003 | 0.07 | | 7.0 | -0.26 |
| ***2.5*** | 2.534 | 2.633 | 2.506 | 2.18 | 2.567 | 2.484 | 0.18 | | 7.1 | 0.64 |
| ***5*** | 6.209 | 4.634 | 5.816 | 4.869 | 6.333 | 5.572 | 0.78 | | 14.0 | -11.44 |
| ***r^2^*** | 0.962 | 0.997 | 0.987 | 0.989 | 0.963 | **0.979** | **0.02** | | 1.6 |  |
| ***Intercept*** | 0.062 | 0.070 | -0.010 | -0.034 | 0.033 | **0.024** | **0.05** | | 188.0 |  |
| ***Slope*** | 4.067 | 5.166 | 5.381 | 5.525 | 5.502 | **5.128** | **0.61** | | 11.9 |  |

***Table S4.***	*Summary of calibration curve parameters in oral fluid for Linezolid (LZD), PNU-142586, and PNU-142300. Curves were prepared on five separate days by multiple operators, each with six calibration levels and a zero level. For each analyte, the table shows the nominal concentrations, the coefficient of determination (r²), the slope and intercept of the linear regression, and the mean, standard deviation (SD), coefficient of variation (%CV), and mean relative error (%MRE) across days. Data fitted to a linear model (1/x² weighting, origin excluded).*

| ***Analyte:*** | ***LZD*** |  |  |  |  |  |  |  |  |
| --- | --- | --- | --- | --- | --- | --- | --- | --- | --- |
| **Nominal conc.** | ***Curve #1*** | ***Curve #2*** | ***Curve #3*** | ***Curve #4*** | ***Curve #5*** | **Mean** | **SD** | **%CV** | **%MRE** |
| ***0*** | 0.004 | 0 | 0.034 | 0 | 0.003 | 0.008 | 0.01 |  |  |
| ***0.1*** | 0.099 | 0.099 | 0.095 | 0.101 | 0.101 | 0.099 | 0.00 | 2.5 | 1.00 |
| ***0.25*** | 0.263 | 0.252 | 0.272 | 0.245 | 0.247 | 0.256 | 0.01 | 4.5 | -2.32 |
| ***0.5*** | 0.485 | 0.513 | 0.536 | 0.503 | 0.485 | 0.504 | 0.02 | 4.2 | -0.88 |
| ***1*** | 0.962 | 1.034 | 0.967 | 1.003 | 1.017 | 0.997 | 0.03 | 3.1 | 0.34 |
| ***2.5*** | 2.602 | 2.449 | 2.499 | 2.533 | 2.603 | 2.537 | 0.07 | 2.6 | -1.49 |
| ***5*** | 4.936 | 4.815 | 4.586 | 4.955 | 4.884 | 4.835 | 0.15 | 3.1 | 3.30 |
| ***r^2^*** | 0.998 | 0.999 | 0.993 | 1.000 | 0.999 | **0.998** | **0.00** | 0.3 |  |
| ***Intercept*** | -0.312 | 0.052 | -2.679 | 1.392 | -0.146 | **-0.339** | **1.47** | -434.6 |  |
| ***Slope*** | 78.981 | 64.114 | 79.631 | 72.646 | 78.335 | **74.74** | **6.56** | 8.8 |  |
|  |  |  |  |  |  |  |  |  |  |
| ***Analyte:*** | ***PNU-142586*** | |  |  |  |  |  |  |  |
| **Nominal conc.** | ***Curve #1*** | ***Curve #2*** | ***Curve #3*** | ***Curve #4*** | ***Curve #5*** | **Mean** | **SD** | **%CV** | **%MRE** |
| ***0*** | 0.005 | 0.013 | 0.036 | 0 | 0.001 | 0.011 | 0.01 |  |  |
| ***0.1*** | 0.101 | 0.099 | 0.095 | 0.101 | 0.102 | 0.100 | 0.00 | 2.8 | 0.40 |
| ***0.25*** | 0.256 | 0.254 | 0.278 | 0.252 | 0.24 | 0.256 | 0.01 | 5.4 | -2.40 |
| ***0.5*** | 0.461 | 0.53 | 0.52 | 0.473 | 0.497 | 0.496 | 0.03 | 6.0 | 0.76 |
| ***1*** | 0.973 | 0.956 | 0.96 | 0.98 | 0.963 | 0.966 | 0.01 | 1.0 | 3.36 |
| ***2.5*** | 2.631 | 2.438 | 2.441 | 2.578 | 2.638 | 2.545 | 0.10 | 3.9 | -1.81 |
| ***5*** | 5.111 | 5.031 | 4.797 | 5.135 | 5.041 | 5.023 | 0.13 | 2.7 | -0.46 |
| ***r^2^*** | 0.997 | 0.998 | 0.994 | 0.998 | 0.998 | **0.997** | **0.00** | 0.2 |  |
| ***Intercept*** | -0.074 | -0.159 | -0.500 | 0.170 | -0.013 | **-0.115** | **0.25** | -214.3 |  |
| ***Slope*** | 15.320 | 12.496 | 13.869 | 13.896 | 15.151 | **14.146** | **1.15** | 8.1 |  |
|  |  |  |  |  |  |  |  |  |  |
| ***Analyte:*** | ***PNU-142300*** | |  |  |  |  |  |  |  |
| **Nominal Conc.** | ***Curve #1*** | ***Curve #2*** | ***Curve #3*** | ***Curve #4*** | ***Curve #5*** | **Mean** | **SD** | **%CV** | **%MRE** |
| ***0*** | 0.014 | 0.013 | 0.033 | 0 | 0.013 | 0.015 | 0.01 |  |  |
| ***0.1*** | 0.101 | 0.099 | 0.093 | 0.102 | 0.101 | 0.099 | 0.00 | 3.7 | 0.80 |
| ***0.25*** | 0.25 | 0.255 | 0.287 | 0.243 | 0.248 | 0.257 | 0.02 | 6.8 | -2.64 |
| ***0.5*** | 0.472 | 0.52 | 0.538 | 0.485 | 0.486 | 0.500 | 0.03 | 5.5 | -0.04 |
| ***1*** | 0.954 | 0.966 | 0.96 | 0.986 | 0.968 | 0.967 | 0.01 | 1.2 | 3.32 |
| ***2.5*** | 2.568 | 2.471 | 2.497 | 2.568 | 2.631 | 2.547 | 0.06 | 2.5 | -1.88 |
| ***5*** | 5.309 | 4.984 | 4.428 | 5.135 | 5.024 | 4.976 | 0.33 | 6.7 | 0.48 |
| ***r^2^*** | 0.997 | 0.999 | 0.986 | 0.999 | 0.999 | **0.996** | **0.01** | 0.5 |  |
| ***Intercept*** | -0.207 | -0.224 | -0.550 | 0.072 | -0.239 | **-0.230** | **0.22** | -95.9 |  |
| ***Slope*** | 15.019 | 16.652 | 16.480 | 15.753 | 18.384 | **16.457** | **1.26** | 7.6 |  |

***Table S5.***	*Summary of calibration curve parameters in plasma for Linezolid (LZD), PNU-142586, and PNU-142300. Curves were prepared on five separate days by multiple operators, each with six calibration levels and a zero level. For each analyte, the table shows the nominal concentrations, the coefficient of determination (r²), the slope and intercept of the linear regression, and the mean, standard deviation (SD), coefficient of variation (%CV), and mean relative error (%MRE) across days. Data fitted to a linear model (1/x² weighting, origin excluded).*

| ***Analyte:*** | | ***LZD*** | |  | |  | |  | |  | |  | |  | |  | |  |  |
| --- | --- | --- | --- | --- | --- | --- | --- | --- | --- | --- | --- | --- | --- | --- | --- | --- | --- | --- | --- |
| **Nominal conc.** | ***Curve #1*** | | ***Curve #2*** | | ***Curve #3*** | | ***Curve #4*** | | ***Curve #5*** | | **Mean** | | **SD** | | **%CV** | | **%MRE** | | |
| ***0*** | 0.002 | | 0 | | 0 | | 0 | | 0 | | 0.000 | | 0.00 | |  |  |  |  |  |
| ***0.1*** | 0.099 | | 0.099 | | 0.106 | | 0.1 | | 0.101 | | 0.101 | | 0.00 | | 2.9 | | -1.00 | | |
| ***0.25*** | 0.255 | | 0.258 | | 0.213 | | 0.248 | | 0.241 | | 0.243 | | 0.02 | | 7.4 | | 2.80 | | |
| ***0.5*** | 0.509 | | 0.492 | | 0.488 | | 0.52 | | 0.514 | | 0.505 | | 0.01 | | 2.8 | | -0.92 | | |
| ***1*** | 1.069 | | 1.051 | | 1.073 | | 1.026 | | 1.041 | | 1.052 | | 0.02 | | 1.9 | | -5.20 | | |
| ***2.5*** | 2.267 | | 2.344 | | 2.377 | | 2.205 | | 2.469 | | 2.332 | | 0.10 | | 4.4 | | 6.70 | | |
| ***5*** | 5.003 | | 5.034 | | 5.457 | | 5.317 | | 4.869 | | 5.136 | | 0.24 | | 4.7 | | -2.72 | | |
| ***r^2^*** | 0.996 | | 0.998 | | 0.988 | | 0.994 | | 0.999 | | **0.995** | | **0.00** | | 0.4 | |  | | |
| ***Intercept*** | -0.081 | | 0.125 | | 4.204 | | 0.012 | | 0.285 | | **0.909** | | **1.85** | | 203.2 | |  | | |
| ***Slope*** | 72.020 | | 72.838 | | 75.736 | | 72.334 | | 76.461 | | **73.878** | | **2.06** | | 2.8 | |  | | |
|  |  | |  | |  | |  | |  | |  | |  | |  | |  | | |
| ***Analyte:*** | ***PNU-142586*** | | | |  | |  | |  | |  | |  | |  | |  | | |
| **Nominal conc.** | ***Curve #1*** | | ***Curve #2*** | | ***Curve #3*** | | ***Curve #4*** | | ***Curve #5*** | | **Mean** | | **SD** | | **%CV** | | **%MRE** | | |
| ***0*** | 0.018 | | 0 | | 0 | | 0 | | 0 | | 0.004 | | 0.01 | |  |  |  |  |  |
| ***0.1*** | 0.102 | | 0.104 | | 0.109 | | 0.103 | | 0.105 | | 0.105 | | 0.00 | | 2.6 | | -4.60 | | |
| ***0.25*** | 0.244 | | 0.233 | | 0.203 | | 0.238 | | 0.218 | | 0.227 | | 0.02 | | 7.3 | | 9.12 | | |
| ***0.5*** | 0.471 | | 0.454 | | 0.435 | | 0.464 | | 0.483 | | 0.461 | | 0.02 | | 3.9 | | 7.72 | | |
| ***1*** | 0.995 | | 1.005 | | 1.03 | | 0.991 | | 1.032 | | 1.011 | | 0.02 | | 1.9 | | -1.06 | | |
| ***2.5*** | 2.394 | | 2.398 | | 2.393 | | 2.333 | | 2.503 | | 2.404 | | 0.06 | | 2.6 | | 3.83 | | |
| ***5*** | 5.537 | | 5.758 | | 6.177 | | 5.804 | | 5.38 | | 5.731 | | 0.30 | | 5.3 | | -14.62 | | |
| ***r^2^*** | 0.995 | | 0.989 | | 0.967 | | 0.989 | | 0.992 | | **0.986** | | **0.01** | | 1.1 | |  | | |
| ***Intercept*** | -0.218 | | 0.318 | | 0.501 | | 0.020 | | 0.089 | | **0.142** | | **0.28** | | 195.0 | |  | | |
| ***Slope*** | 12.145 | | 10.471 | | 10.370 | | 10.182 | | 12.064 | | **11.046** | | **0.97** | | 8.8 | |  | | |
|  |  | |  | |  | |  | |  | |  | |  | |  | |  | | |
| ***Analyte:*** | ***PNU-142300*** | | | |  | |  | |  | |  | |  | |  | |  | | |
| **Nominal Conc.** | ***Curve #1*** | | ***Curve #2*** | | ***Curve #3*** | | ***Curve #4*** | | ***Curve #5*** | | **Mean** | | **SD** | | **%CV** | | **%MRE** | | |
| ***0*** | 0 | | 0 | | 0 | | 0.001 | | 0 | | 0.000 | | 0.00 | |  |  |  |  |  |
| ***0.1*** | 0.1 | | 0.104 | | 0.108 | | 0.1103 | | 0.105 | | 0.105 | | 0.00 | | 3.7 | | -5.46 | | |
| ***0.25*** | 0.244 | | 0.238 | | 0.202 | | 0.228 | | 0.221 | | 0.227 | | 0.02 | | 7.2 | | 9.36 | | |
| ***0.5*** | 0.518 | | 0.439 | | 0.47 | | 0.502 | | 0.475 | | 0.481 | | 0.03 | | 6.4 | | 3.84 | | |
| ***1*** | 1.075 | | 0.975 | | 1.05 | | 1.029 | | 1.02 | | 1.030 | | 0.04 | | 3.6 | | -2.98 | | |
| ***2.5*** | 2.217 | | 2.458 | | 2.321 | | 2.256 | | 2.519 | | 2.354 | | 0.13 | | 5.5 | | 5.83 | | |
| ***5*** | 5.137 | | 5.839 | | 5.955 | | 5.607 | | 5.435 | | 5.595 | | 0.33 | | 5.8 | | -11.89 | | |
| ***r^2^*** | 0.994 | | 0.986 | | 0.974 | | 0.990 | | 0.992 | | **0.987** | | **0.01** | | 0.8 | |  | | |
| ***Intercept*** | 0.059 | | 0.268 | | 0.266 | | -0.008 | | 0.087 | | **0.134** | | **0.13** | | 93.7 | |  | | |
| ***Slope*** | 12.610 | | 12.851 | | 12.438 | | 12.214 | | 13.046 | | **12.632** | | **0.33** | | 2.6 | |  | | |

***Figure S1.***	*Representative chromatogram of a processed blank VAMS sample (without analytes or IS).*

***Figure S2.***	*Representative chromatogram of a processed VAMS sample spiked with Linezolid (LZD), PNU-142586, and PNU-142300 at 5 µg/mL, and the IS (LZD-d3) at its working concentration.*

***Figure S3.*** *Representative chromatogram of a processed blank oral fluid sample (without analytes or IS).*

***Figure S4.***	*Representative chromatogram of a processed oral fluid sample spiked with Linezolid (LZD), PNU-142586, and PNU-142300 at 5 µg/mL, and the IS (LZD-d3) at its working concentration.*

***Figure S5.*** *Representative chromatogram of a processed blank plasma sample (without analytes or IS).*

***Figure S6.***	*Representative chromatogram of a processed plasma sample spiked with Linezolid (LZD), PNU-142586, and PNU-142300 at 5 µg/mL, and the IS (LZD-d3) at its working concentration.*

***Table S6.***	*Autosampler stability of Linezolid (LZD), PNU-142586, and PNU-142300 in processed samples after 72 h at 6ºC. Results are expressed as mean percentage difference (%Difference) from freshly injected QC samples (n=3 for each concentration level).*

| ***Matrix*** | ***Analyte*** | ***% Difference*** | | |
| --- | --- | --- | --- | --- |
|  |  | ***Low QC*** (0.2 µg/mL) | ***Medium QC*** (2 µg/mL) | ***High QC*** (4 µg/mL) |
| ***VAMS*** | *LZD* | -3.6 | -1.5 | -0.5 |
|  | *PNU-142586* | -3.0 | -2.4 | -5.2 |
|  | *PNU-142300* | 0.9 | 3.8 | 1.4 |
| ***Oral Fluid*** | *LZD* | 1.1 | 1.4 | 1.5 |
|  | *PNU-142586* | 0.0 | 3.6 | 5.4 |
|  | *PNU-142300* | 0.9 | 0.2 | -0.1 |

***Table S7.***	*Experimental concentrations (mean ± SD, n = 3) of Linezolid (LZD), PNU-142586, and PNU-142300 measured in VAMS devices loaded with whole blood at three hematocrit (Hct) levels (Low: 0.25; Medium: 0.45; High: 0.7) and three nominal concentration levels (0.2, 2, and 4 µg/mL).*

| ***Analyte*** | ***Nominal concentration*** | ***Experimental concentration (µg/mL)*** | | |
| --- | --- | --- | --- | --- |
|  |  | ***Low Hct*** | ***Medium Hct*** | ***High Hct*** |
| ***LZD*** | *0.2 µg/mL* | 0.228±0.003 | 0.243±0.012 | 0.220±0.015 |
|  | *2 µg/mL* | 2.052±0.153 | 2.116±0.135 | 1.960±0.076 |
|  | *4 µg/mL* | 4.197±0.036 | 3.999±0.189 | 3.777±0.275 |
|  | | | | |
| ***PNU-142586*** | *0.2 µg/mL* | 0.238±0.005 | 0.215±0.009 | 0.180±0.022 |
|  | *2 µg/mL* | 2.160±0.242 | 2.069±0.114 | 1.605±0.044 |
|  | *4 µg/mL* | 4.560±0.185 | 3.943±0.176 | 3.073±0.142 |
|  | | | | |
| ***PNU-142300*** | *0.2 µg/mL* | 0.210±0.018 | 0.221±0.015 | 0.199±0.021 |
|  | *2 µg/mL* | 1.853±0.185 | 2.005±0.117 | 1.879±0.024 |
|  | *4 µg/mL* | 4.057±0.116 | 3.910±0.158 | 3.553±0.231 |

***Table S8.***	*Influence of hematocrit (Hct) levels (Low: 0.25; Medium: 0.45; High: 0.7) on assay precision expressed as coefficient of variation (CV%) for Linezolid (LZD), PNU-142586, and PNU-142300 at low, medium, and high concentration levels in VAMS samples.*

| ***Analyte*** | ***Concentration*** | ***CV%*** | | | |
| --- | --- | --- | --- | --- | --- |
|  |  | ***Low Hct*** | ***Medium Hct*** | ***High Hct*** | ***Mean*** |
| ***LZD*** | *0.2 µg/mL* | 1.5 | 5.1 | 6.7 | 6.2 |
|  | *2 µg/mL* | 7.5 | 6.4 | 3.9 | 6.3 |
|  | *4 µg/mL* | 0.9 | 4.7 | 7.3 | 6.2 |
|  | | | | |  |
| ***PNU-142586*** | *0.2 µg/mL* | 2.0 | 3.9 | 12.1 | 13.4 |
|  | *2 µg/mL* | 11.2 | 5.5 | 2.7 | 15.0 |
|  | *4 µg/mL* | 4.1 | 4.5 | 4.6 | 17.2 |
|  | | | | |  |
| ***PNU-142300*** | *0.2 µg/mL* | 8.7 | 6.9 | 10.8 | 9.0 |
|  | *2 µg/mL* | 10.0 | 5.9 | 1.3 | 6.8 |
|  | *4 µg/mL* | 2.9 | 4.0 | 6.5 | 7.0 |

***Table S9.***	*Individual linezolid (LZD), PNU-142586, and PNU-142300 concentrations measured in plasma, VAMS loaded with venous whole blood (VAMS_WB), capillary VAMS, and oral fluid (OF). Each row corresponds to a paired sample obtained from the same patient. Suspected outliers were excluded before averaging replicates to yield a single value per sample, analyte, and matrix.*

| ***LZD*** | | | | ***PNU-142586*** | | | | ***PNU-142300*** | | | | |  |
| --- | --- | --- | --- | --- | --- | --- | --- | --- | --- | --- | --- | --- | --- |
| ***VAMS*** | ***VAMS_WB*** | ***PLASMA*** | ***OF*** | ***VAMS*** | ***VAMS_WB*** | ***PLASMA*** | ***OF*** | | ***VAMS*** | ***VAMS_WB*** | ***PLASMA*** | ***OF*** | |
| 1.77 | 2.10 | 2.12 | 2.31 | 1.53 | 1.99 | 2.50 | 0.04 | | 0.31 | 0.40 | 0.70 | 0.20 | |
|  | 2.13 | 1.88 |  |  | 7.27 | 7.73 |  | |  | 1.63 | 2.40 |  | |
| 1.59 | 1.87 | 1.85 | 2.64 | 1.26 | 2.32 | 1.77 | 0.05 | | 0.35 | 0.57 | 0.58 | 0.21 | |
| 0.99 | 0.02 | 0.02 |  | 0.12 | 0.16 | 0.12 |  | | 0.02 | 0.02 | 0.03 |  | |
| 12.40 | 9.01 | 5.96 |  | 2.06 | 2.36 | 2.95 |  | | 1.23 | 1.13 | 1.79 |  | |
| 0.12 | 0.01 | 0.01 | 0.02 | 0.18 | 0.35 | 0.14 | 0.02 | | 0.08 | 0.13 | 0.07 | 0.02 | |
| 1.87 | 0.96 | 1.03 |  | 2.55 | 4.17 | 3.45 |  | | 0.45 | 0.66 | 0.76 |  | |
| 7.17 | 8.22 | 8.81 |  | 4.77 | 2.81 | 3.75 |  | | 1.51 | 1.68 | 3.04 |  | |
| 3.36 | 5.58 | 5.16 |  | 1.43 | 1.94 | 2.35 |  | | 0.44 | 0.66 | 1.08 |  | |
| 10.98 | 14.83 |  |  | 31.29 | 32.45 |  |  | | 14.56 | 14.51 |  |  | |
| 7.11 | 15.72 | 12.14 | 11.52 | 1.49 | 4.30 | 5.04 | 0.49 | | 1.39 | 3.51 | 5.09 | 2.16 | |
|  | 7.56 | 6.73 | 7.73 |  | 17.42 | 24.16 | 0.05 | |  | 12.41 | 21.77 | 0.36 | |
| 2.19 | 2.50 | 2.10 | 3.09 | 1.78 | 2.52 | 1.51 | 0.15 | | 0.45 | 0.59 | 0.47 | 0.36 | |
| 2.98 | 2.77 | 2.57 | 3.31 | 2.98 | 2.98 | 2.30 | 0.08 | | 0.90 | 0.93 | 0.98 | 0.08 | |
| 2.29 | 2.03 | 1.83 |  | 8.77 | 10.34 | 3.58 |  | | 4.92 | 6.13 | 1.88 |  | |
|  | 1.84 | 2.00 | 3.95 |  | 3.14 | 4.00 | 0.02 | |  | 0.64 | 1.11 | 0.07 | |
| 12.09 | 13.39 | 13.46 |  | 9.15 | 5.29 | 12.76 |  | | 3.92 | 3.68 | 6.95 |  | |
| 10.04 | 12.14 | 11.04 |  | 1.10 | 1.40 | 2.44 |  | | 0.92 | 1.03 | 2.24 |  | |
| 2.29 | 2.02 | 1.81 | 4.78 | 4.31 | 6.53 | 4.69 | 0.01 | | 1.07 | 1.66 | 1.63 | 0.04 | |
| 2.58 | 2.60 | 2.30 | 2.30 | 2.78 | 3.18 | 2.33 | 0.05 | | 1.08 | 1.22 | 1.30 | 0.08 | |
| 1.13 | 1.11 | 0.97 | 1.89 | 2.40 | 2.91 | 2.18 | 0.24 | | 0.37 | 0.41 | 0.44 | 0.25 | |
| 0.81 | 0.83 | 0.80 | 0.85 | 2.36 | 4.96 | 3.77 | 0.07 | | 0.34 | 0.73 | 0.81 | 0.07 | |
| 5.64 | 4.52 | 4.32 | 5.44 | 1.75 | 2.44 | 3.71 | 2.10 | | 0.88 | 0.83 | 1.73 | 1.81 | |
| 15.26 | 13.86 | 11.41 | 19.01 | 6.28 | 6.31 | 7.25 | 0.24 | | 3.53 | 3.39 | 5.27 | 1.14 | |
| 2.11 | 1.58 | 1.44 | 2.13 | 4.70 | 3.87 | 4.49 | 0.01 | | 1.21 | 0.97 | 1.35 | 0.03 | |
| 2.50 | 2.27 | 2.03 |  | 1.65 | 1.46 | 1.09 |  | | 0.41 | 0.39 | 0.42 |  | |
| 2.29 | 1.98 | 1.70 |  | 4.04 | 3.85 | 3.97 |  | | 0.80 | 0.70 | 0.97 |  | |
| 4.28 | 3.96 | 3.29 | 3.49 | 3.09 | 2.54 | 3.19 | 0.06 | | 1.87 | 1.46 | 2.28 | 0.16 | |
| 2.23 | 2.70 | 2.13 | 3.20 | 2.00 | 2.67 | 2.90 | 0.05 | | 0.76 | 1.01 | 1.44 | 0.12 | |
|  | 15.39 | 13.25 |  |  | 3.46 | 4.47 |  | |  | 3.38 | 5.35 |  | |
| 13.69 | 15.35 | 13.41 | 14.31 | 6.44 | 7.45 | 8.58 | 0.73 | | 3.67 | 4.61 | 6.86 | 1.23 | |
| 3.70 | 3.58 | 3.19 | 4.17 | 3.00 | 2.83 | 3.16 | 0.06 | | 1.05 | 1.02 | 1.55 | 0.20 | |
| 10.68 | 13.77 | 11.05 | 12.66 | 3.85 | 3.53 | 6.00 | 0.08 | | 1.67 | 1.58 | 3.95 | 0.29 | |
| 6.19 | 6.03 | 4.84 | 14.89 | 24.18 | 23.16 | 24.30 | 0.27 | | 22.40 | 21.23 | 28.87 | 1.10 | |
| 6.75 | 6.54 | 5.16 |  | 13.80 | 12.91 | 14.89 |  | | 4.66 | 4.36 | 6.86 |  | |

***Table S10.***	*Median concentrations [interquartile range, IQR], minimum–maximum values, and standard deviations (SD) of linezolid (LZD) and its metabolites PNU-142586 and PNU-142300 in VAMS loaded with capillary blood (VAMS, n = 31), VAMS loaded with whole blood (VAMS_WB, n = 35), plasma (n = 34), and oral fluid collected with Salivette® (ORAL_FLUID, n = 21). All concentrations are expressed in µg/mL.*

| ***Analyte*** | ***Matrix*** | ***Median [IQR]*** | ***Min–Max*** | ***SD*** |
| --- | --- | --- | --- | --- |
| ***LZD*** | VAMS | 2.98 [2.11–7.17] | 0.12–15.26 | 4.34 |
|  | VAMS_WB | 2.77 [1.98–9.01] | 0.01–15.72 | 5.22 |
|  | Plasma | 2.44 [1.83–7.25] | 0.01–13.46 | 4.33 |
|  | ORAL_FLUID | 3.49 [2.31–9.62] | 0.02–19.01 | 5.32 |
| ***PNU-142586*** | VAMS | 2.78 [1.65–4.77] | 0.12–31.29 | 6.77 |
|  | VAMS_WB | 3.19 [2.44–6.31] | 0.16–32.45 | 6.62 |
|  | Plasma | 3.65 [2.34–5.28] | 0.12–24.30 | 5.71 |
|  | ORAL_FLUID | 0.06 [0.044–0.241] | 0.01–2.10 | 0.46 |
| ***PNU-142300*** | VAMS | 1.05 [0.443–1.87] | 0.02–22.40 | 4.59 |
|  | VAMS_WB | 1.03 [0.656–3.39] | 0.02–21.23 | 4.49 |
|  | Plasma | 1.59 [0.800–4.23] | 0.03–28.87 | 5.94 |
|  | ORAL_FLUID | 0.20 [0.074–0.732] | 0.02–2.16 | 0.63 |

***Figure S7.***	*Histograms with overlay density curves (gray) of Linezolid (LZD) concentrations measured in each matrix.*

***Figure S8.***	*Boxplots of Linezolid (LZD) concentrations (µg/mL) across different biological matrices. Boxes represent the interquartile range (IQR) and horizontal lines indicate the. Sample size (n) is indicated in each box.*

***Figure S9.***	*Q-Q plots of Linezolid (LZD) concentrations for each matrix. Theoretical quantiles are plotted against observed values. Diagonal line (gray) represents expected distribution under normality.*

***Figure S10.***	*Histograms with overlay density curves (gray) of PNU-142586 concentrations measured in each matrix.*

***Figure S11.***	*Boxplots of PNU-142586 concentrations (µg/mL) across different biological matrices. Boxes represent the interquartile range (IQR) and horizontal lines indicate the. Sample size (n) is indicated in each box.*

***Figure S12.***	*Q-Q plots of PNU-142586 concentrations for each matrix. Theoretical quantiles are plotted against observed values. Diagonal line (gray) represents expected distribution under normality.*

***Figure S13.***	*Histograms with overlay density curves (gray) of PNU-142300 concentrations measured in each matrix.*

***Figure S14.***	*Boxplots of PNU-142300 concentrations (µg/mL) across different biological matrices. Boxes represent the interquartile range (IQR) and horizontal lines indicate the. Sample size (n) is indicated in each box.*

***Figure S15.***	*Q-Q plots of PNU-142300 concentrations for each matrix. Theoretical quantiles are plotted against observed values. Diagonal line (gray) represents expected distribution under normality.*

***Table S11.***	*Method comparison results for Linezolid (LZD), PNU-142586, and PNU-142300, showing sample size (n), statistical difference tests, Passing–Bablok regression parameters (95% CI), and Pearson’s correlation coefficients. VAMS: volumetric absorptive microsampling devices loaded with capillary blood; VAMS_WB: VAMS loaded with whole blood; ORAL_FLUID: Oral fluid collected with Salivette®. All experimental values were included in the calculation.*

| ***Analyte*** | ***Comparison*** | ***n*** | ***Test (p-value)*** | ***Diff. Sig.?*** | ***Passing-Bablok*** | | ***Pearson’s r (p-value)*** | ***Corr. Sig.?*** |
| --- | --- | --- | --- | --- | --- | --- | --- | --- |
|  |  |  |  |  | ***Intercept (95% CI)*** | ***Slope (95% CI)*** |  |  |
| ***LZD*** | VAMS_WB vs Plasma | 34 | Wilcoxon (3.19e-06) | Yes | -0.095 (-0.261 to 0.001) | 1.167 (1.1192 to 1.245) | 0.989 (8.45e-28) | Yes |
|  | VAMS vs Plasma | 30 | Wilcoxon (0.100) | No | 0.331 (-0.166 to 0.723) | 1.005 (0.870 to 1.282) | 0.907 (5.10e-12) | Yes |
|  | Plasma vs ORAL_FLUID | 21 | Wilcoxon (1.34e-05) | Yes | 0.464 (-0.520 to 0.704) | 1.1526 (1.043–1.703) | 0.884 (1.05e-07) | Yes |
|  | VAMS vs VAMS_WB | 31 | Wilcoxon (0.650) | No | 0.542 (0.764 to 0.988) | 0.861 (0.764–0.988) | 0.923 (1.41e-13) | Yes |
| ***PNU-142586*** | VAMS_WB vs Plasma | 34 | Wilcoxon (0.078) | No | 0.164 (-0.258 to 0.669) | 0.848 (0.715–0.959) | 0.922 (9.19e-15) | Yes |
|  | VAMS vs Plasma | 30 | Wilcoxon (0.017) | Yes | -0.052 (-0.844 to 0.361) | 0.930 (0.742 to 1.056) | 0.949 (1.61e-05) | Yes |
|  | Plasma vs ORAL_FLUID | 21 | Wilcoxon (9.54e-07) | Yes | 0.030 (-0.028 to 0.081) | 0.010 (-0.002 to 0.029) | 0.024 (0.917) | No |
|  | VAMS vs VAMS_WB | 31 | Paired t (0.226) | No | -0.401 (-0.996 to -0.096) | 1.046 (0.904 to 1.187) | 0.982 (1.93e-22) | Yes |
| ***PNU-142300*** | VAMS_WB vs Plasma | 34 | Wilcoxon (5.14e-06) | Yes | 0.126 (0.043 to 0.209) | 0.616 (0.560 to 0.665) | 0.967 (1.30e-20) | Yes |
|  | VAMS vs Plasma | 30 | Wilcoxon (3.24e-06) | Yes | 0.048 (-0.103 to 0.172) | 0.618 (0.513 to 0.736) | 0.973 (2.19e-19) | Yes |
|  | Plasma vs ORAL_FLUID | 21 | Wilcoxon (2.86e-06) | Yes | 0.056 (-0.071 to 0.181) | 0.046 (0.010–0.168) | 0.320 (0.157) | No |
|  | VAMS vs VAMS_WB | 31 | Wilcoxon (0.258) | No | -0.076 (-0.163 to -0.009) | 1.046 (0.924 to 1.090) | 0.993 (9.31e-29) | Yes |

***Table S12.***	*Mean percentage prediction error (MPPE), mean absolute percentage error (MAPE), mean absolute error (MAE), and number of paired samples (n) obtained for each analyte and comparison. VAMS: volumetric absorptive microsampling devices loaded with capillary blood; VAMS_WB: VAMS loaded with whole blood.*

| **Analyte** | **Comparison** | **Including all values** | | | | **Excluding values < 0.1 µg/mL** | | | |
| --- | --- | --- | --- | --- | --- | --- | --- | --- | --- |
|  |  | ***MPPE (%)*** | ***MAPE (%)*** | ***MAE (µg/mL)*** | ***n*** | ***MPPE (%)*** | ***MAPE (%)*** | ***MAE (µg/mL)*** | ***n*** |
| ***LZD*** | VAMS_WB vs PLASMA | 13.2 | 14.5 | 0.8 | 34 | 12.8 | 14.3 | 0.8 | 32 |
|  | VAMS vs PLASMA | 247.6 | 257.48 | 1.13 | 30 | 15.10 | 25.71 | 1.17 | 28 |
|  | VAMS vs VAMS_WB | 206.3 | 222.8 | 11.6 | 31 | 0.8 | 18.5 | 1.2 | 29 |
| ***PNU-142586*** | VAMS_WB vs PLASMA | 7.0 | 33.6 | 13.7 | 34 | 7.0 | 33.6 | 1.4 | 34 |
|  | VAMS vs PLASMA | -6.8 | 29.0 | 1.1 | 30 | -6.8 | 29.0 | 1.1 | 30 |
|  | VAMS vs VAMS_WB | -10.0 | 24.8 | 0.9 | 31 | -10.0 | 24.8 | 0.9 | 31 |
| ***PNU-142300*** | VAMS_WB vs PLASMA | -17.4 | 37.4 | 13.6 | 34 | -20.2 | 36.1 | 1.4 | 32 |
|  | VAMS vs PLASMA | -27.1 | 39.0 | 1.16 | 30 | -28.6 | 40.1 | 1.2 | 28 |
|  | VAMS vs VAMS_WB | -10.7 | 18.3 | 0.3 | 31 | -10.2 | 18.3 | 0.3 | 29 |

***Table S13.***	*Bland-Altman comparison results. VAMS: volumetric absorptive microsampling devices loaded with capillary blood; VAMS_WB: VAMS loaded with whole blood.*

| **Analyte** | **Comparison** | **Bland–Altman** | | |
| --- | --- | --- | --- | --- |
|  |  | **Mean Diff.** | **Upper LoA** | **Lower LoA** |
| ***LZD*** | VAMS_WB vs Plasma | 0.7531 | 2.7787 | -1.2725 |
|  | VAMS vs Plasma | 0.3238 | 4.0519 | -3.4042 |
|  | VAMS vs VAMS_WB | -0.5457 | 3.5393 | -4.6308 |
| ***PNU-142586*** | VAMS_WB vs Plasma | -0.4919 | 3.9377 | -4.9215 |
|  | VAMS vs Plasma | -0.5120 | 2.5451 | -3.5692 |
|  | VAMS vs VAMS_WB | -0.2862 | 2.2396 | -2.8119 |
| ***PNU-142300*** | VAMS_WB vs Plasma | -1.1118 | 3.6216 | -5.8451 |
|  | VAMS vs Plasma | -1.0271 | 2.2411 | -4.2953 |
|  | VAMS vs VAMS_WB | -0.1376 | 0.9691 | -1.2442 |
